# Anthocyanins: Modified New Technologies and Challenges

**DOI:** 10.3390/foods12071368

**Published:** 2023-03-23

**Authors:** Yang Lin, Cong Li, Lejuan Shi, Lixia Wang

**Affiliations:** 1 Department of Food Science and Technology, Zhejiang University of Technology, Hangzhou 310014, China; 2 Zhejiang Sci-Tech University Shaoxing Academy of Biomedicine Co., Ltd., Shaoxing 312000, China; 3 Changshan Agriculture Development Center, Changshan 324200, China

**Keywords:** anthocyanins, molecular modification, physical modification, application progress

## Abstract

Anthocyanins are bioactive compounds belonging to the flavonoid class which are commonly applied in foods due to their attractive color and health-promoting benefits. However, the instability of anthocyanins leads to their easy degradation, reduction in bioactivity, and color fading in food processing, which limits their application and causes economic losses. Therefore, the objective of this review is to provide a systematic evaluation of the published research on modified methods of anthocyanin use. Modification technology of anthocyanins mainly includes chemical modification (chemical acylation, enzymatic acylation, and formation of pyran anthocyanidin), co-pigmentation, and physical modification (microencapsulation and preparation of pickering emulsion). Modification technology of anthocyanins can not only increase bioavailability and stability of anthocyanin but also can improve effects of anthocyanin on disease prevention and treatment. We also propose potential challenges and perspectives for diversification of anthocyanin-rich products for food application. Overall, integrated strategies are warranted for improving anthocyanin stabilization and promoting their further application in the food industry, medicine, and other fields.

## 1. Introduction

Anthocyanins, a category of phenolic compounds, are one of the most important water-soluble pigments in nature [1]. They are widely found in the cell sap of plant flowers, fruits, stems, leaves, and root organs, and they are responsible for the red, purple, or blue coloration of fruits and flowers [2,3,4]. In the food industry, anthocyanins are used as pigments for pastry, candies, coloring of drinks, jellies and jelly-type desserts, etc. Commission regulation (EU) No 231/2012 of 9 March 2012 set specifications for food additives, including anthocyanin, with the E-163 code [5]. Anthocyanins have been recognized as food colorants by several countries, such as Australia, New Zealand, and some EU countries, with the code E-163 [4,6].

**Figure 1:**
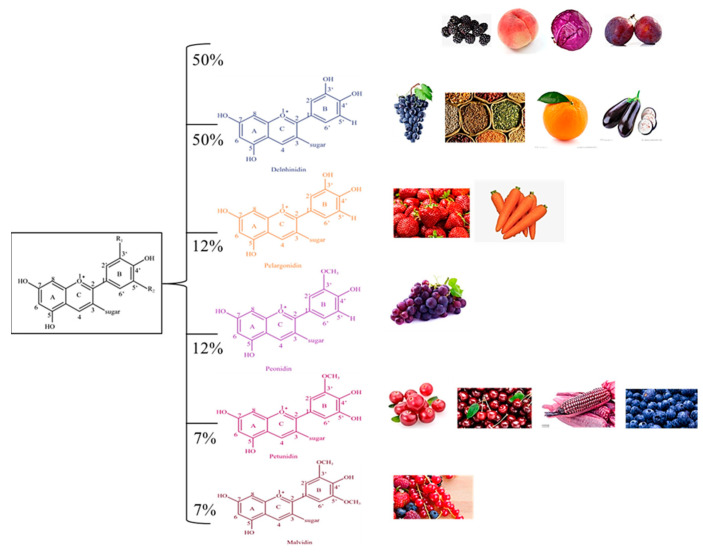
The structure and composition of natural anthocyanins.

Anthocyanins are flavonoid derivatives formed by glycosidic bonds between anthocyanidins with a core structure of 3, 5, 7-tri-hydroxyl-2-phenylbenzo-pyran cation and aglycones at the C3 site [7,8,9]. The different chemical structures of anthocyanins arise from the position and number of hydroxyl groups on the molecule, the degree of methylation, the nature and number of sugar moieties attached to the aglycone, and the position of the attachment [10,11]. More than 700 kinds of anthocyanins with 30 different core structures have been identified [12]; anthocyanins, delphiniums, pelargonidin, peonidin, petunidin, and malvidin are the six typical types of anthocyanins [13]. Their structure and proportion in nature are shown in Figure 1.

Anthocyanins have various potential therapeutic effects on diabetes [14], colon cancer [15,16], cardiovascular disease [17], atherosclerosis [18], etc. According to the latest research, anthocyanins could be a potential dietary supplement to prevent neurodegenerative diseases [19].

In addition, recent research has reported that anthocyanins have important antioxidant and antimicrobial properties [20,21,22]. Moreover, physiological functions of anthocyanins are widely used in the field of food packaging. Anthocyanins are pH-sensitive substances, and their molecular structures and colors change with pH variation [23]. In addition, intelligent colorimetric packaging films can be prepared through loading anthocyanins to polysaccharides, proteins, and other biopolymers, and these show diverse colors in different acid–base environments. In this way, it is possible to indicate and monitor the freshness of packaged products in real time [24]. Meanwhile, anthocyanins are active components with antioxidant and antimicrobial abilities, which can prolong food shelf-life [3,23] and can be used as antibacterial agents in the field of food packaging [8,25,26]. Anthocyanins are highly reactive towards reactive oxygen species [18,27].

The low stability of anthocyanins is the primary obstacle to their commercial application as colorants in the food industry [1,13,28,29]. Indeed, numerous environmental factors, including pH, temperature, light, pressure, oxygen, enzymes, and metallic ions can damage anthocyanins [30,31]. However, adverse conditions for anthocyanin maintenance are inevitable through complex food processing such as thermal processing and fermentation [29,30,32]. Therefore, improvement of the stability of anthocyanins is an urgent problem to be solved.

Additionally, despite the beneficial properties of anthocyanins, their effectiveness at preventing or treating diseases is limited by their low bio-accessibility and bioavailability [7]. The Food and Drug Administration (FDA) defines the term bioavailability as “the rate and extent to which the active ingredient or moiety is absorbed and becomes available at the site of action”. An analysis of anthocyanins’ bio-accessibility and bioavailability was performed by analyzing blood and urine anthocyanin concentrations following ingestion of foods containing large amounts of anthocyanins [33]. The bioavailability of anthocyanins is one of the lowest among flavonoids; it is estimated at less than 1–2% [34]. After anthocyanins are released from plant cell vacuoles, the contraction and interactions with other food and biological components, such as carbohydrates, fiber, proteins, enzymes, or other polyphenols, may affect their bio-accessibility. Meanwhile, the low bioavailability of anthocyanins may also stem from the instability caused by pH changes as well as microbial and enzyme degradation during gastrointestinal passage [35,36,37]. 

**Figure 2:**
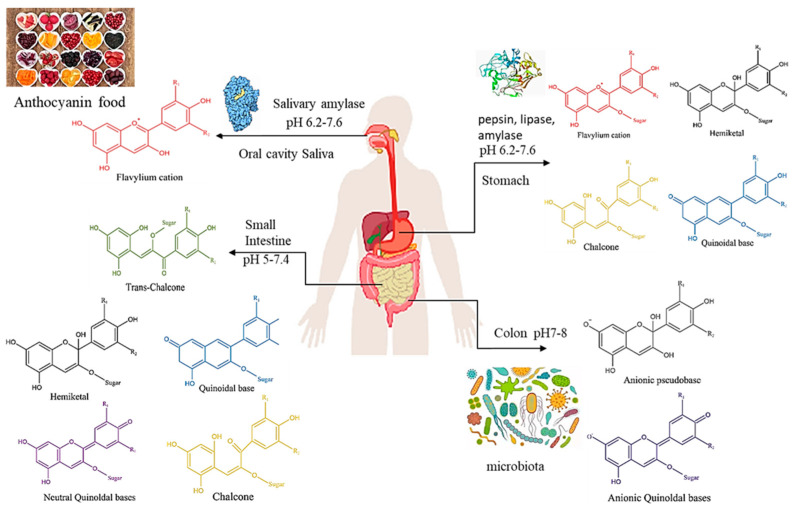
Schematic representation of anthocyanin degradation and absorption in different regions of the human gastrointestinal tract [7].

The degradation and absorption pathways of anthocyanins in the human body are shown in Figure 2. First, the oral cavity contains many salivary amylases at pH 7.4, which might result in some early anthocyanin degradation [38]. Under the acidic conditions prevailing in the gastric compartment, anthocyanins are in the positively charged flavylium form, where anthocyanins are quickly absorbed (approximately 25%) [39]. The pepsin, lipase, and amylase in the stomach may interact with anthocyanins to produce stable complexes [7,34]. Therefore, the rapid absorption of anthocyanins in the stomach and the formation of complexes affect their metabolism and reduce their bioavailability. Moreover, anthocyanins are extensively metabolized in the gut [40]. In the small intestine, where the pH is close to 7, anthocyanins may be present in a mixture of structural forms (flavylium, quinoidal bases, hemiketal, and chalcone), and quinoidal and/or hemiketal forms could predominate [41]. Hemiketal forms are more susceptible to oxidative degradation than flavylium cations, which may lead to their breakdown to yield smaller phenolic products such as phenolic acids [40]. The human colon is home to a diverse and large number of microorganisms, with counts reaching 10^12^–10^14^ CFU/mL [34]. These microbial groups can extensively catabolize anthocyanins, thereby contributing to the increase in bioavailability. The bioavailability of anthocyanins is closely related to human health, and promoting the slow release of anthocyanins in the intestines and making them metabolized and absorbed by microorganisms can improve their bioavailability.

Therefore, this review will focus on innovative and advanced strategies in terms of the mechanisms and recent advances for enhancing anthocyanin stabilization. In addition, we comprehensively evaluated the properties of modified anthocyanins and their role in disease prevention and treatment as well as proposed potential challenges and perspectives for application of anthocyanin-rich products. In conclusion, this review aims to provide guidance for improving the potential and application scope of anthocyanins as value-adding pigments and raw materials in the food industry.

## 2. Chemical Modification of Anthocyanins

### 2.1. Modification of Acyl

#### 2.1.1. Chemical Acylation

Chemically, anthocyanins are glycosylated, polyhydroxy, or polymethyl derivatives of a 2-phenyl-1-benzopyrylium moiety [42]. In fact, the majority of all known anthocyanins are acylated [43,44]. Acylated anthocyanins impart desirable color and stability to vegetables and fruits, such as radishes, red potatoes, red cabbage, black carrots, and purple sweet potatoes [45]. 

In nature, the hydroxyl groups (AOHs) of the substituted glycosyls (i.e., the sugar moieties) of anthocyanins are typically acylated with organic acids via ester bonds, which is referred to as anthocyanin glycosyl acylation, to yield acylated anthocyanins [44]. Lauric acid reacts with the primary hydroxyl group of glucoside and removes a molecule of water to obtain acylated derivatives [46]. Anthocyanin glycosyl acylation is performed mainly through hydrophobic and “π-π” interactions between the acyl donor and anthocyanin molecule [13]. Acyl substituents are commonly bound to the C3 sugar or esterified to the 6-OH (or less frequently to the 4-OH) group of the sugars [46]. π-stacking interactions between phenolic nuclei are promoted by anthocyanins acylated by hydroxycinnamic acid (HCA) residues. The diacylated anthocyanins maintain a higher percentage of cationic and neutral-colored forms at equilibrium under mildly acidic conditions. Therefore, acylation can protect the anthocyanin chromophore from water attack (result of π stacking of acyl-anthocyanins) [47]. The protective effect of acylation on anthocyanin increases with the number of acyl groups [48]. Organic acids are the source of acyl donors for acylated anthocyanins. The organic acids acylating the sugar moieties of anthocyanins include aliphatic and aromatic (phenolic) acids. Table 1 shows the acylation modification of anthocyanins by different organic acids. 

Chemical acylation is unable to carry out the reaction at a specific position of the hydroxyl group, and it is easy to bind or shield some of the main active phenolic hydroxyl groups of anthocyanins, thereby affecting the acylation. Cruz et al. [49] have reported that the chemical acylation of a pure malvidin-3-*O*-glucoside (Mv3glc) using stearoyl chloride in anhydrous acetonitrile yielded the stearic acid derivative, which was not regioselective and produced a complex mixture of mono-, di-, and tri-ester derivatives. 

**Table 1:** Acylation modification of anthocyanins by organic acids.

Organic Acid Type	Acyl Donor Type	Acyl Donor Structure	References
aliphatic	acetic acid	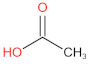	[2]
	palmitic acid	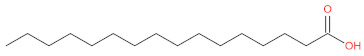	[46,50]
	octanoic acid	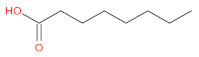	[51]
	lauric Acid	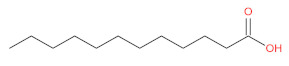	[52]
	saturated fatty acids of different chain lengths	—	[53]
aromatic (phenolic) acids	caffeic acid	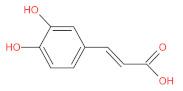	[43,47,54,55]
	p-coumaric acid	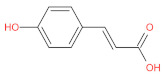	[43,54,55,56]
	cinnamic acid	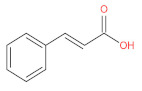	[46,56,57,58]
	p-hydroxycinnamic acids	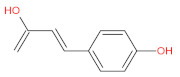	[47,48]
	p-hydroxybenzoic acids	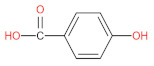	[47]
	ferulic	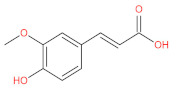	[55]

#### 2.1.2. Enzymatic Acylation

The enzymatic acylation reaction usually occurs in a specific position of the anthocyanin structure through hydrophobic and “π-π” interactions [13,51,57]. Enzymatic acylation mainly includes the following two types: direct acylation and transesterification. In the directly acylated reaction, fatty acids or phenolic acids are used as acyl donors in organic solvents at low water activity, and the water byproduct is removed by molecular sieving. In the transesterification reaction, fatty acids or aromatic carboxylic acid vinyl esters are used as acyl donors, but acyl donors need to be synthesized for the reaction in advance. 

Acylation is the primary way to increase the polarity, molecular size, and to change the spatial structure of anthocyanins. Therefore, active site of acylation, acyl types, and numbers can interfere with the effects of acylation. Since enzymes are enzymatic acylation catalysts, the degree of acylation is influenced by enzyme acylation reaction conditions such as acyl donor types, enzymes, and reaction media (Table 2).

First of all, common acyl donors mainly include aliphatic, aromatic (phenolic) acids and fatty acid esters. The acylation rates of fatty acids with different chain lengths as acyl donors showed different acylation rates, ranging from 21% to 40%. Among them, caprylic acid showed the best acylation effect and the highest acylation rate [51]. The study by Liu et al. [43] showed that the acylation degrees of blueberry anthocyanins with coumaric acid and caffeic acid were 5.38% and 5.68%, separately. It is precisely due to the different structures of the acyl donors, especially the distribution of the hydroxyl groups on the aromatic ring, that the acylation rate of the reactions of methyl benzoate, methyl salicylate, and methyl cinnamate are different [61]. When methyl salicylate and methyl benzoate are used as acyl donors, the conversion rates can reach 84.26% and 91%, respectively [13,63].

Acting as a catalyst, acylase contributes to high specificity and catalytic efficiency for targets on the particular groups in the structure to carry out acylation at a mild condition [64]. Moreover, the conversion rate of acylated products is affected by enzyme concentration [50,57,65]. Free lipase from Candida antarctica and immobilized lipase Novozymes 435 are two commonly used enzymes to catalyze the acylation of anthocyanins. For esterification of primary alcohols, Candida antarctica lipase B (CAL-B), which can be used to prepare pure products, has great regioselectivity [59]. For instance, the malvidin-3-glucoside-oleic acid ester and Delphinidin 3-*O*-sambubioside-lipophilic acid ester were regioselectively synthesized by CAL-B, and the acylation rates exceeded 20% [60]. Novozymes 435 is an immobilized preparation of heat-stabilized lipase. It has broad substrate specificity and can promote the esterification reaction between primary alcohols [46], secondary alcohols, and carboxylic acids in a wide range [43]. Compared with CAL-B-catalyzed anthocyanin acylation, Novozymes 435-catalyzed anthocyanin acylation had a higher conversion rate when fatty acids were used as acyl donors [52,53]. Novozymes 435 can also catalyze the transesterification of anthocyanins. In addition, the steps of chemical acylation are complex and cumbersome, so activated acyl donors are often prepared and used for acylation [63].

The nature of the reaction medium can affect the acylation product conversion rate and the catalytic power of enzymes [56]. Generally, the higher the solubility of anthocyanins in the solvents, the higher the conversion rate of acylated products. In the acylation of anthocyanin, tert-butanol, tert-amyl alcohol, acetone, acetonitrile, pyridine, and 2-methyl-2-butanol are commonly used as solvents [53,57]. Cyanidin-3-glucoside (C3G) has greater zero-time solubility in pyridine and a higher acylation conversion rate (70.3%) compared to 2M2B (59.5%). Although the zero-time solubility of Cyanidin-3-glucoside (C3G) in tert-butanol is less than 40%, as the stirring time increases, the solubility increases, and the conversion rate reaches 85.7% [61]. Therefore, it is extremely critical to select appropriate enzymatic acylation conditions to obtain ideal anthocyanin acylation products. 

Generally, chemical acylation of anthocyanin is feasible to perform. The progress of chemical acylation is usually limited by external environmental factors. Compared with enzymatic acylation, chemical acylation is not region-selective, which may lead to undesirable functionalization of hydroxyl groups. Enzymatic acylation of anthocyanins with high yield can prepare special acylated anthocyanins with high stability under special conditions. Taken together, enzymatic acylation is considered as a more effective method than chemical acylation with regards to enhancing anthocyanin stabilization in application [64]. Purification and removal of unexpected byproducts from acylated anthocyanin is a problem that needs to be overcome for the production of acylated anthocyanin on a food industry scale.

### 2.2. Pyran Anthocyanin

The history of pyran anthocyanins dates back to the 1990s, when a new class of pigments was detected in red wine filtrates [66]. At present, many pyran anthocyanins have been separated and identified in fermented fruit wine or fruit juice beverages. The main types of pyran anthocyanins include the following five: hydroxyphenyl-pyran anthocyanins, vitisins pyran anthocyanins, vinylflflavanole-pyranoanthocyanins, portisins, and rosacyanin B [67]. Its basic structure is based on the proanthocyanin structure, and the fourth D-ring is formed by a cycloaddition reaction between the C4 and C5 hydroxyl groups of anthocyanins [67].

Pyrananthin and anthocyanins differ in physiochemical properties, such as color and stability [68,69]. The new compounds named pyrananthin, first discovered in port wine, present a charming and rare turquoise blue color under acidic conditions. The new pyran ring protects anthocyanin against the nucleophilic attack of water, which hinders the formation of the carbinol base, resists the affinity attack of acid sulfite, and enhances stability. In addition, pyran anthocyanin-flavanol derivatives exhibit complete resistance to sulfur dioxide bleaching and enhanced stability during storage [69]. In the process of wine brewing, a small amount of oxygen is added to micro-oxidize anthocyanins, forming pyran anthocyanins, which stabilize and enhance the color of the wine [70,71].

However, the application of pyran anthocyanin in food industry is limited by the time-consuming nature of the process and low conversion rates.

## 3. Physical Modification of Anthocyanins

### 3.1. Microencapsulation

**Figure 3:**
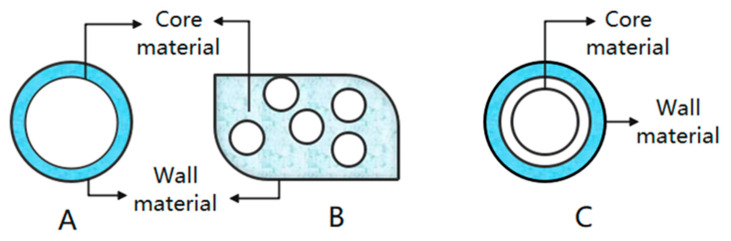
Several common types of microencapsulation. (**A**): single capsule; (**B**): microcapsule polymer; (**C**): multiple encapsulation.

Microencapsulation is a new and rapidly developing technology that can be utilized for the incorporation and immobilization of biologically active compounds within or on solid particles (microspheres) or liquid vesicles. Microencapsulation can protect and stabilize the biologically active compounds susceptible to environmental factors, such as curcumin, quercetin, and anthocyanin [72]. Figure 3 shows several common types of microencapsulation. Anthocyanins, a sensitive biologically active substance, are encapsulated in microcapsules to maintain their stability and prolong their shelf life [73]. The type of wall material and microencapsulation methods have the greatest influence on the stability and embedding efficiency of anthocyanin microencapsulation [74]. Core material release properties and microcapsule stability are two key factors in selecting wall materials [75,76]. The method of microencapsulation chosen minimizes environmental factors that harm anthocyanins.

#### 3.1.1. Wall Material Type

The stability and embedding efficiency of microencapsulation mainly depend on the types of wall materials [74]. The basic characteristics of the main wall require emulsifying, film-forming, water-solubility, and high stability, and it must not react with the core material [77]. Anthocyanins are hydrophilic colorants that are particularly compatible with water-based gel formulations. Therefore, maltodextrin [77,78], gum Arabic [54], starch, and its derivative gums [79] are commonly used water-based gel formulations for encapsulating anthocyanins [76]. 

Single-wall materials do not meet all the requirements needed to improve encapsulation properties [64]. Therefore, the microencapsulation of anthocyanins usually involves composite wall materials to achieve a better encapsulation effect. The encapsulation efficacy of anthocyanins encapsulated with maltodextrin/modified maize starch in different ratios of wall material was between 93.1% and 97.4% [79]. Akhavan et al. [77] prepared microcapsules containing anthocyanins with maltodextrin and gum Arabic as the wall materials, and the microencapsulation efficiency (ME) of anthocyanins was as high as 92.83%, which is due to the cross-linking interaction between the carboxymethyl starch (CMS)/xanthine gum (XG) combination and anthocyanins; the encapsulation efficiency of CMS/XG-encapsulated anthocyanin is above 96% [58].

In addition to polysaccharide-based wall materials, proteins, especially whey protein isolate and soy protein isolate, are widely used as wall materials for encapsulating anthocyanins. The research of Michael et al. [28] showed that thermally induced whey-protein-based microcapsules suitable for encapsulating anthocyanin-rich bilberry extract can be generated from whey protein solutions. Whey protein was used to prepare cherry peel anthocyanin microcapsules. The encapsulation efficiency reached 70.30 ± 2.20%, which contained 31.95 ± 0.65 mg CGE/100 g DW anthocyanins [80]. Mansour et al. [81] successfully encapsulated red raspberry anthocyanins using a combination of soy protein isolate and gum Arabic. 

#### 3.1.2. Microencapsulation Method

The preparation methods of microcapsules mainly include spray drying, freeze drying, vacuum drying, and drum drying (Table 3). Microcapsule technology also includes vibrating nozzles, centrifugal extrusion, and crystallization. Among these, spray drying and freeze drying are commonly used for preparing anthocyanin microcapsules.

The spray-drying method can produce a powdery anthocyanin particle with improved storage stability, easier handling, and minimized transportation [82]. Anthocyanin microcapsules with maltodextrin as the wall material were prepared by spray drying technology, and the encapsulation efficiency reached 96.7%. Furthermore, the anthocyanin microcapsules prepared by spray-drying technology showed good storage stability of anthocyanin [83,84,85].

Freeze drying facilitates dehydration of the frozen mixtures of anthocyanins and wall materials by sublimation under vacuum and low temperatures, which maintains its chemical structure and reduces the risk of undesirable changes [7]. The retention of anthocyanin prepared by freeze-drying technology was higher than 76% after 90 days of storage under UV light [86]. When compared to other methods of anthocyanin encapsulation, freeze-dried double emulsion (FDE) microcapsules had higher total anthocyanin and total phenolic contents [21]. 

Furthermore, studies have shown that encapsulation of anthocyanins prepared by the combination of freeze drying and spray drying also show great properties. For example, Fredes et al. [87] combined spray-drying with freeze-drying technology to prepare anthocyanin microcapsules, resulting in improved anthocyanin retention and bio-accessibility of yogurt before consumption.

Because it takes a long time and because the manufacturing mode is discontinuous, we do not recommend freeze drying production encapsulation of anthocyanins on a food industrial scale. For spray drying, high temperature adversely affects encapsulated anthocyanins. Taken together, anthocyanin encapsulations have not been well applied in industrial production. 

**Table 3:** Study on the microencapsulation of anthocyanins.

Source	Wall Materials	Proportion	Encapsulation Efficiency	Encapsulation	References
grape seed	maltodextrin (MD), mesquite gum (MG), and zein (Z)	44% MG-56% Z wall: core material is 2:1 in a 2% (*w*/*v*) total solids dispersion	85%	spray drying	[26]
		34% MD-66% Z wall: core material is 2:1 in a 2% (*w*/*v*) total solids dispersion	82%		
juçara fruits	maltodextrin and gum Arabic	maltodextrin and gum Arabic in a 1:1 proportion; wall material: core material 2:3	83.69%	freeze drying	[54]
blueberry	carboxymethyl starch (CMS)/xanthan gum (XG)	CMS/XG: 30/1, 60/1, 90/1, 120/1, 150/1, *w*/*w*%	over 96%	freeze drying	[58]
purple rice bran	modified glutinous rice starch	anthocyanin extract 40 mg cyanidin-3-glucoside/L and 6.01% modified starch	94.22%	spray drying	[74]
blueberries	inulin, gum Arabic, and maltodextrin DE20	maltodextrin DE20, hi-maize gum Arabic, and inulin 6.66%/5%	ranged from 96.80 to 98.83%	spray drying	[76]
barberry	maltodextrin and gelatin	wall material content and anthocyanin load of 24.54% and 13.82%, respectively	92.83%	spray drying	[77]
Iranian borage	maltodextrin (MD) and modified maize starch (MMS)	MD/MMS: 1/0, 1/0.25, 1/0.5, 1/1, *w*/*w*%; wall material: core material 1:4	93.1 and 97.4%	spray drying	[79]
sour cherries skins	whey protein isolate and gum Arabic	5% whey proteins isolate and 2% gum Arabic	70.30 ± 2.20%	freeze drying	[80]
red raspberry	soy protein isolate (SPI) and gum Arabic (AG)	different concentrations of anthocyanin (0.025%, 0.05%, and 0.075%); the concentration of SPI or AG was 5%, *w*/*v,* while for a combination of SPI + AG, 2.5% *w*/*v* for each was used	ranged from 93.05% to 98.87%	freeze drying	[81]
red cabbage	maltodextrin dextrose equivalent 20 and Arabic gum (AG)	MD20:AG 20:80	ranged from 93.65 ± 1.80 to 98.85 ± 0.32%	drum drying	[84]
Cornelian cherry	whey protein isolates, inulin, and chitosan	WPI, chitosan, and inulin in a ratio of 2:1:1 (w:w:w)	89.16 ± 1.23%	freeze drying	[88]
grape skins	soy phosphatidylcholine vesicles with the addition of pectin, acacia gum, and whey protein isolates	soy lecithin (100 mg mL^−1^), pectin (1 mg mL^−1^), acacia gum (1 mg mL^−1^), and whey protein isolates (1 mg mL^−1^)	ranged from 81 to 96%	freeze drying	[89]
mulberry	alginate/chitosan beads	freeze-dried beads (100 mg) loaded with mulberry-extracted solution containing anthocyanin (40 mL)	/	freeze drying	[90]
red cabbage	maltodextrin and Arabic gum	maltodextrin (25, 35, and 50 g), Arabic gum (25, 15, and 0 g), and critic acid (1 g) were dispersed in 100 mL solution	67%	spray drying	[91]
saffron	ß-glucan and ß-cyclodextrin	ß-glucan	45%	spray drying	[92]
		ß-cyclodextrin	63.25%		
grape skin	sodium alginate	sodium alginate: anthocyanin extract of grape skin 1:15	75%	spray drying	[93]
blueberry	chitosan and cellulose nanocrystal (CNC)	chitosan (0.1% *w*/*v*) pH 2.6 and 20 mL of 0.025–2.5% (*w*/*v*) CNC	94%	ionic gelation	[94]

### 3.2. Pickering Emulsion

In recent years, pickering emulsions and their applications have attracted much attention due to their ease of preparation and enhanced stability [95]. Emulsions are conventionally stabilized by a combination of electrostatic stabilization, reduced interfacial tension, and steric stabilization by means of surfactants or soluble macromolecules [95]. The particles adsorbed at the oil–water interface form a physical barrier, which can block the interface interaction and droplet contact through volume exclusion [96]. 

Pickering emulsion is primarily used as a delivery system for nutraceuticals such as curcumin and resveratrol [97,98,99,100,101]. In the field of food science, the application of food-grade particles endows the pickering emulsions with a broader prospect [96]. Food-grade particles for pickering emulsion applications are mainly divided into six categories: polysaccharide particles, protein-based particles, complex particles, flavonoid particles, food-grade wax, and fat crystals [99,102]. Pickering emulsion can avoid the damage of anthocyanins by external environmental factors and is also an effective carrier for protecting and transporting anthocyanins. Different food-grade particles loaded with anthocyanins and the characteristics of pickering emulsion stabilized by composite nanoparticles are summarized in Table 4. 

The formation of anthocyanin nanoparticles is based on the interaction between anthocyanins and the encapsulating material, which helps to prepare a stable pickering emulsion. Electrostatic interactions, covalent interactions, hydrogen bonding, and van der Waals interactions are all common interactions between anthocyanins and wall materials. Anthocyanins loaded by polysaccharide-based nanoparticles doped anthocyanins within the complex nanocarriers, and the encapsulation rate of anthocyanins reached 66.68% [103]. The covalent interaction between anthocyanins and protein, which allows protein peptide chains to be unfolded, could significantly promote the formation of emulsion network structures [104,105,106]. The particle size of anthocyanin microcapsules is smaller than that of unloaded nanoliposomes, which might be due to the interaction of anthocyanin with lipid acyl chains and alteration of acyl chain order [20]. Furthermore, the self-assembly method is also used to prepare stable nanoparticles. Stable vesicles that encapsulated anthocyanins were formulated based on the self-assembling of L-α-phosphatidylcholine (PC) and mannosylerythritol lipid-A (MEL-A) in a manner of weak or non-cooperative interactions [22]. Pectin with net negative charge and lysozyme with net positive charge were also used to prepare composite nanoparticles through the self-assembly method [107]. 

Double-layer pickering emulsion, which is used to load and transport anthocyanins, showed a high encapsulation rate and a slow-release effect of anthocyanin [108,109,110]. Double emulsion usually has either water-in-oil-in-water (W/O/W) or oil-in-water-in-oil (O/W/O) form, whereby the dispersed droplets contain smaller droplets of a different phase, essentially an emulsion in an emulsion. The presence of two interfaces means that two emulsifiers are required to stabilize the inner primary and outer secondary emulsions [92]. Double emulsions could retain the structural integrity and high encapsulation stability of anthocyanins (95%), which provides a potential route for anthocyanin delivery [111].

Pickering emulsion can overcome damage of anthocyanins during processing, storage, and human digestion, and it can be performed on an industrial scale. With the continuous development of pickering emulsion technology, we can soon expect more common use of this technology for anthocyanin applications, even in the industry.

**Table 4:** Different food-grade particles loaded with anthocyanins and the characteristics of pickering emulsion stabilized by composite nanoparticles.

Particle Type	Material	Source	Particle Size	Encapsulation Efficiency	In Vitro Digestion Experiment Results and Other Functional Characteristics	Reference
Liposome particles	mainly composed of lecithin, cholesterol, and Tween 80	cranberry	Average particle size of nanoparticles (53.8 ± 1.8 nm)	91.1% ± 1.7%	Retention rate from the anthocyanin-loaded nanoliposomes and unencapsulated anthocyanins were 88.19% and 73.20%, respectively.	[20]
	the self-assembling of L-α-phosphatidylcholine (PC) and mannosylerythritol lipid-A (MEL-A)	cyanidin 3-*O*-glucoside	Anthocyanins are encapsulated in vesicles with an average diameter between 200 and 700 nm, and the core size is less than 500 nm	54.9% ± 1.6%	During the gastric digestion, the release rate of anthocyanins was kept below 20%; in the intestinal tract, the release contents of anthocyanins were increased to 53.3 ± 3.3% within 30 min.	[22]
Composite particles	gelatin(GEL)and chitosan (CS)	red raspberry pomace	When the ratio of GEL to CS is 6:4, the smallest nanoparticles are formed	83.81%	Anthocyanins have suitable long-term storage capacity at room temperature, with a retention rate of ~50% after 15 d.	[36]
	chitosan and pectin	bilberry	When the mass ratio of chitosan/pectin/anthocyanin is 1:1:3, the nanocarrier is a well-dispersed sphere with a diameter of about 150 nm	66.68%	After 12 h digestion, the release rate of anthocyanins from complex nanocarriers in gastric juice was 26%, and that the release rate in intestine juice was 56%.	[103]
	chitosan hydrochloride (CHC), carboxymethyl chitosan (CMC)	cyanidin-3-*O*-glucoside	Under the best conditions, the nanocomposite particles have a better particle size (178.1 nm)	44.0%	These ACN-loaded CHC/CMC nanocomplexes protected the anthocyanins from degradation by storage at different conventional temperature, various ascorbic acid (AA) concentrations, varying pH, and white fluorescent light.	[102]
	chitosan hydrochloride (CHC), carboxymethyl chitosan (CMC) and whey protein isolate (WPI)	*Lycium ruthenicum* murray	The nanocomposite loaded with anthocyanin has a good particle size (332.20 nm)	60.70%	The ACN-CHC/CMC-WPI nanocomplexes showed a slow-release of anthocyanins, releasing only 53.5% of the ACNs. The cumulative ACN release from ACN-CHC/CMC-WPI nanocomplexes (9.4%) was significantly lower than from the unencapsulated form (20.8%).	[14]
	pectin and lysozyme	blackberry	The particle size of the nanocomposite is 198.5 nm	73%	The particles were stable in different pH ranges according to the size and zeta potential measurements. In the simulated gastrointestinal fluid, the ACN in ACN-CHC/CMC-WPI is more stable over time, and the release rate is slower.	[107]
Protein particles	isolated soy protein (SPI)	black rice	With the increase of anthocyanins concentration (0–0.2%), the particle size gradually decreased (186–675 nm)	94.1%	The pickering emulsion exhibited significantly lowered LH and MDA contents by up to 85.9% and 81.1%, respectively, indicating its superior oxidative stability.	[104]
Polysaccharides particles	octenylsuccinate quinoa starch (OSQS)	bilberry	130 μm to 25 μm,	95%	The encapsulation stability of anthocyanins in double emulsions decreased from 92.9% to 86.2% and 93.4% to 86.6% for the volume ratio of (W_1_/O): W_2_ = 6:4 and 5:5 during gastric digestion, respectively. The anthocyanin retention in the double emulsions decreased significantly to 42.1% and 37.6% during small intestine digestion for the volume ratio of (W_1_/O): W_2_ = 6:4 and 5:5, respectively.	[109]
	amylopectin	commercial products	About 100 nm	84%	After 2 h simulated intestinal digestion, 29.21% of the anthocyanins were retained.	[112]

## 4. Small Molecule Co-Pigmentation Agent

In food science, the interaction of co-pigmentation is very important to improve product color and acceptance [113]. Sari et al. (2012) described co-pigmentation as a phenomenon in which anthocyanins and other colorless organic compounds, or metallic ions, form molecular or complex associations, generating a change or an increment in the color intensity [114]. Molecular co-color is a unique property of anthocyanins that does not exist in other polyphenols. 

The interaction with the co-pigment constructs a tangible mask for the anthocyanin, which not only shades the functional moieties of anthocyanin molecules and reduces their accessibility and activity to adverse reactions but also constitutes a great steric hindrance to the attack of destroyers of anthocyanins [115]. Polyphenols, flavonoids, peptides, amino acids, and organic acids are often applied to co-pigments, which interact with anthocyanin molecules by van der Waals forces, hydrogen bonds, hydrophobic forces, and ionic interactions [116].

### 4.1. Co-Pigmentation Effect of Polyphenols and Flavonoids on Anthocyanins

Polyphenolics show a good co-pigmentation effect due to their extended π-π conjugated system [117]. Organic acids, aromatic acyl groups, or flavonoids (or some combination thereof) and the chromophore of anthocyanins are covalently linked to achieve co-color through loose intermolecular interactions. Colorless flavonoids or other phenolic compounds interact with anthocyanins through weak hydrophobic forces [118]. The co-pigments with more methoxyl groups or hydroxyl groups interact with anthocyanins to form more stable complexes [117]. Hydroxycinnamic acids generally had better co-pigmentation performances than hydroxybenzoic acids [119]. Since phenolic acids are weaker cofactors than flavonoids with an extensive—conjugated system, flavanols such as quercetin-3-rutinoside (ruin) are the most efficient co-pigments [117,120]. 

Different phenolic substances have different co-pigmentation effects on anthocyanins. The studies of co-pigmentation of black chokeberry anthocyanins with 10 kinds of phenolic co-pigments showed different co-pigmentation effects, which manifested as high color and color shifts. Compared with vanillin, epigallocatechin gallate, and protocatechualdehyde, the half-life for anthocyanin color fading in the model beverage increased from 2.9 to 6.7 days with green tea extract [121]. 

### 4.2. Co-Pigmentation Effect of Peptides and Amino Acids on Anthocyanins

Amino acids and peptides also have co-pigmenting effects with anthocyanins through hydrogen bonds, hydrophobic interactions, and van der Waals forces. Chung et al. [122] found that the hydrogen bonding interaction between L-tryptophan and anthocyanin increased the average half-life of anthocyanin from two days to six days. Li et al. [123] found that the physicochemical stabilities of cyanidin-3-*O*-glucoside (C3G) in alkali conditions, Cu^2+^ ions, and at a high temperature were significantly enhanced after combination with silk fibroin peptide (SFP). Van der Waals and hydrogen bonding were found between anthocyanins and lactoferrin (LF) and LF-derived peptides, which enhanced the color stability of anthocyanins [124]. Based on the hydrophobic force and hydrogen bonding interactions between anthocyanins and whey protein (WP), adding natural WP (10 mg/mL) can prolong anthocyanin half-life by about 1–2 times [125].

### 4.3. Co-Pigmentation Effect of Organic Acids on Anthocyanins

Organic acid is a small molecule substance that can also show co-pigmentation effects with anthocyanins through covalent connection or loose intermolecular interactions. Co-pigmentation leads to the hyperchromic effect arising from the absorbance enhancement in the visible range and a positive shift in maximum absorbance wavelength (bathochromic shift), which indicates an increase in color intensity [114,126]. The reactions of anthocyanins and cofactors are spontaneously exothermic. Compared with gallic acid, ellagic acid has a higher negative Gibbs free energy, which leads to a greater co-pigmentation effect on anthocyanins.

### 4.4. Co-Pigmentation Effect of Metal Ions on Anthocyanins

Color and stability of anthocyanins were enhanced by the addition of multivalent ions, such as Mg^2+^, Fe^2+^, Fe^3+^, and Al^3+^. Hydroxyl groups on the B-ring of anthocyanins bind with metal cations to form a stable metal–anthocyanin complex [127]. The complexation process transforms red flavylium cations into purple–blue quinoidal base anions. This transformed group can then stack with other flavylium cation molecules to form stable metal-coordinated complexes [128]. This phenomenon can improve the stability of the anthocyanin while intensifying its color. Anthocyanins, flavones, and metal cations can form complicated supermolecules. Shiono et al. found that blue colors of corn flower pigments are complicated supermolecules composed of anthocyanins, flavones, and metal cations.

Co-pigmentation is easy to perform to protect anthocyanin during the practical processing of food. The addition of co-pigments increases the stability and can even change the bioactivity of anthocyanins. Co-pigmentation techniques are commonly practiced in the food industry to adjust food color to retain or reconstitute natural color intensity or to create new hues.

## 5. Improved Performances of Modified Anthocyanins

Instability of anthocyanins leads to their easy degradation, reduced bioactivity, and color fading in food processing, which limits their application and causes economic losses. Therefore, it is urgent and necessary to investigate suitable methods to maintain and improve anthocyanin stability for development, production, and storage anthocyanin-rich products [64]. According to the different principles of the method and technology used to modify anthocyanins, they can be roughly divided into two categories: chemical modification and physical modification. The approach of chemical modification focuses on the improvement of anthocyanin structure [129,130], while the physical modification is to encapsulate the anthocyanin molecules to better resist degradation caused by external environmental factors [131,132]. In addition, the co-pigmentation reaction of anthocyanins with small molecules can enhance and stabilize the color of anthocyanins [118,133]. No matter which modification method is selected, the purpose is to improve the stability (storage stability and gastrointestinal digestion stability), lipophilicity, and antioxidant effects of anthocyanins to thereby improve their bioavailability and promote their further application in the food industry.

### 5.1. Stability Performances of Modified Anthocyanins

#### 5.1.1. Storage Stability Performances 

Storage stability is a crucial standard for using anthocyanins as food colorants [63]. However, anthocyanins are very unstable during processing and storage. In particular, the degradation caused by high temperature, light, and ascorbic acid limits their potential applications in the food industry [134,135]. Thus, preventive measures must be implemented for anthocyanins to increase their stability during storage. 

First, anthocyanins are extremely susceptible to environmental temperature during the storage process [136]. In particular, an increase in temperature conferred an active equilibrium shift of anthocyanins tending to colorless chalcone and pseudo base formation [64]. Acylation plays a significant role in improvement of anthocyanin thermostability through “π-π” interactions between the acyl residues and the anthocyanin nucleus. Acylation protects the anthocyanin molecules from nucleophilic attack [13,53]. Anthocyanin complexation with co-pigments via stacking, dipole–dipole interactions, and hydrogen-bonding intermolecular interactions protects anthocyanins from thermal degradation (Table 5) [118,120,134]. The nanocomplex formation through interactions between the encapsulation material and the anthocyanin molecules would maintain the more stable flavylium cation or quinoidal base structures instead of allowing them to hydrate into carbinol or chalcone structures, which also play a role in improving thermal stability [79,137]. For instance, nanocomplex formation through ionic interactions between chitosan derivatives and anthocyanin flavylium cations could prevent the hydration of anthocyanins [102]. Another report also indicated that water-soluble carbohydrates significantly improved the thermal stability of anthocyanins by the reduction of water activity around anthocyanins [138].

On the other hand, anthocyanins are inevitably degraded by light during the process of transportation and storage [103]. The light degradation mechanism of anthocyanin is derived largely from the excitation of the flavylium cation [139]. Therefore, a prominent method of protecting anthocyanins against photodegradation is that they are acylated, which, through intramolecular stacking of the organic acid to the anthocyanidin nucleus, protects the flavylium cation from excitation [55]. Moreover, diacylated anthocyanins are more stable than monoarylated anthocyanins [8]. Another study suggested that due to the conjugated systems between co-pigments and the benzene rings of anthocyanin, new anthocyanins were formed, which increased the light-energy-absorbing and potential electron-donating abilities of the anthocyanin. This also enhanced the photostability of anthocyanins [44]. Additionally, different from the principle of chemical modification to improve photostability, the physical encapsulate system, due to the protective effect of the wall material on anthocyanins, also improves the light resistance of anthocyanins [35,61]. Sodium alginate used as a wall material in anthocyanin microcapsules can greatly improve anthocyanin light stability [93]. Multifunctional films based on chitosan/gum Arabic have excellent photostability and UV barrier properties [140]. For anthocyanins loaded into chitosan hydrochloride/carboxymethyl chitosan nanocomplexes, compared with natural anthocyanin, the color seemed unchanged after storage for six days [108].

The common addition of ascorbic acid could enhance the nutritional quality of commercial beverage products. However, the heat sterilization process in the presence of ascorbic acid would degrade anthocyanins [141]. The reduced stability of anthocyanins by L-ascorbic acid is mainly attributed to the condensation reaction between anthocyanins and L-ascorbic acid [142]. Previous studies reported molecular binding between anthocyanins and co-pigments such as phenolic and water-soluble polysaccharides through hydrogen bonding or hydrophobic interactions, which prevents the condensation reaction between anthocyanin and ascorbic acid, thus significantly improving anthocyanin stability in the presence of ascorbic acid [116,141,143]. For instance, since both whey protein and ascorbic acid compete to interact with anthocyanins, the addition of whey protein would form a whey protein–anthocyanin interaction, thereby decreasing ascorbic-acid-mediated anthocyanin degradation [125]. The formation of anthocyanin–rosmarinic acid–xanthan gum ternary complexes through shielding the highly electrophilic C2 position of the flavylium cation, which is easily attacked by water and subsequently causes chemical degradation, thereby enables chemical protection of anthocyanin chromophores [125].

**Table 5:** Stability performances of modified anthocyanins.

Anthocyanins	Modified Method	Improvement Effect	Reference
red raspberry pomace anthocyanin extracts	microencapsulation	Anthocyanin-loaded β-Lg nanoparticles were more stable in mouth (pH 6.8), simulated gastric (simulated gastric, pH 2), and simulated intestine (simulated intestinal, pH 6.9) by showing higher retention rate (%) than that of unencapsulated anthocyanins.	[36]
blackcurrant (*Ribes nigrum*) anthocyanins	enzymatic acylation	The half-life of the acylated derivatives was higher than that of the corresponding anthocyanins at each selected temperature.	[52]
cyanidin-3-*O*-galactoside	enzymatic acylation	Compared with C3G, the Ea value of the C3G lauric acid conjugate decreased from 46.6 to 45.8 kJ mol^−1^.	[53]
anthocyanin extracts	enzymatic acylation	The kinetic rate constant (k) and half-life parameter indicated that the thermostability of acylated cyanidin glycosides was higher than C3G.	[57]
blueberry anthocyanins	microencapsulation	The stability of anthocyanins was increased to 76.11% after 30-day storage (37 °C) through carboxymethyl starch/xanthan gum	[58]
raspberry anthocyanin	enzymatic acylation	The half-life of cyanidin-3-(6-salicyloyl) glucoside in the same environment was two times higher than that of cyanindin-3-*O*-glucoside.	[63]
vitisin A vitisin B	pyran anthocyanins	Vitisin A (consists of malvidin 3-glucoside) was entirely protected from bleaching by sulfur dioxide, and vitisin B (which is decarboxyvitisin A or malvidin 3-glucoside) showed greater resistance than malvidin 3-glucoside.	[66]
anthocyanin extracts	microencapsulation	The anthocyanins were chiefly retained inside the microparticles in the stomach and were released in the intestine.	[79]
red raspberry anthocyanin	microencapsulation	All microcapsules enhanced the thermal stability of anthocyanins in the temperature range 80–114 °C. Furthermore, anthocyanins were retained (up to 48%) during storage at 37 °C for 60 days.	[81]
blueberry anthocyanins	nanoparticle encapsulation	After 70 days of storage, the preservation rate of free anthocyanins was 85%, while the preservation rate of anthocyanins encapsulated with chitosan and pectin under dark conditions was higher than 96%.	[103]
spinarum fruit anthocyanins extract	emulsions	After thermal processing at 90 °C for 3 min, the retention of anthocyanins was at a maximum (72.24%) for emulsions.	[105]
anthocyanin extract	co-pigmentation	The addition of whey protein (WP) decreased anthocyanin color degradation significantly during the five day storage study at 25 °C in the dark and improved anthocyanins’ half-life significantly.	[109]
anthocyanin extracts	pickering emulsion	When digested in simulated gastric fluid, the starch-based double emulsions could retain the structural integrity and high encapsulation stability of anthocyanin.	[109]
sour cherry anthocyanins	co-pigmentation	Tannic acid, caffeic acid, 4-hydroxybenzoic acid, gallic acid, and malic acid could enhance the color intensity of sour cherry anthocyanins at pH 3.5.	[118]
purple carrot anthocyanins	co-pigmentation	After the addition of L-tryptophan, the average half-life of anthocyanins increased from two days to six days.	[122]
cyanidin-3-*O*-galactoside chloride, cyanidin-3-*O*-arabinoside	co-pigmentation	The hyperchromic effect of ofrosmarinus acid, syringic acid, and catechin were 51.02%, 43.24%, and 39.73%, respectively.	[133]
cyanidin-3-glucoside	chemical acylation	Retention rates of acylated C3G after heating for 10 h at 80, 100, and 120 °C were 83.24, 74.17, and 62.17%, respectively, which is obviously than higher than unacylated C3G.	[141]
cyanidin-3-*O*-glucoside (C3G)	co-pigmentation	ΔE in anthocyanins was reduced by 35.8% and 79.0%, total anthocyanin degradation dropped by 11.1% and 48.2%, and the average t_1/2_ increased 0.15 and 2.25 times, respectively.	[144]

#### 5.1.2. Gastrointestinal Digestion Stability Performances

Considering that dietary anthocyanins positively contribute to human health, it is particularly necessary to promote the digestion and absorption of dietary anthocyanins in the human body [40]. However, the instability of anthocyanins in gastrointestinal digestion, such as the loss of anthocyanins during gastrointestinal digestion, is not conducive to their physiological functions [145,146]. Additionally, the beneficial properties of anthocyanins are mainly dependent on their intestinal absorption and colonic microbial fermentation [145,147,148]. Therefore, not only is there a need to reduce the release ratio, thus minimizing the loss of anthocyanins during gastrointestinal digestion, but there is also a need to promote their targeted release in the intestine and colonic microbiota fermentation. 

Physical encapsulation has been widely adopted as an effective technique to improve the stability of anthocyanins in gastrointestinal digestion and colonic fermentation [94,147,149]. For instance, gum Arabic used for black rice anthocyanin encapsulation aided in delaying the release of anthocyanins during microstimulated gastrointestinal digestion [111]. Modified starch provides targeting properties to double emulsions, protects anthocyanins from gastric digestion, and controls release with starch hydrolysis in intestinal digestion [109]. Anthocyanins encapsulated with cyclodextrins degraded more slowly during intracolonic fermentation than anthocyanins without encapsulation [150]. Moreover, soy protein isolate can interact with anthocyanins, increasing colonic accessibility and delaying anthocyanin release [94].

The stability of digestion and absorption stability in the gastrointestinal tract of anthocyanins are also related to their bioavailability in the human body [151]. Thus, in future research, the molecular mechanisms of anthocyanin absorption need to be fully clarified to improve in vivo digestion, absorption, bioavailability, and bioactivities of anthocyanins through suitable modification methods.

### 5.2. Antioxidant Activity of Modified Anthocyanins

#### 5.2.1. Chemical Oxidation Resistance

The antioxidant properties of anthocyanins are significant for potential new food and nutraceutical applications [61]. The antioxidant capacity of anthocyanins depends on its structure [152]. Previous studies have found that the antioxidant activity of ACNs is mainly determined by the number of phenolic hydroxyl groups in the B-ring of the parent nucleus, C6-C3-C6 framework [153]. Therefore, the structural modification of anthocyanins helps to improve its chemical-based antioxidant capacity, thereby providing ideas for its antioxidant application in functional foods.

According to the literature, the strong antioxidant capacity of anthocyanins is due to the fact that they contain multiple phenolic hydroxyl groups, which can react with free radicals to generate stable semiquinone radicals, which interrupts the oxidation chain reaction [60,154]. The acylation of anthocyanins with organic acids adds additional phenol-type hydroxyl functions to the overall structure, which enhances the antioxidant activity of the product [155]. However, the antioxidant activity of acylated anthocyanins was affected by the characteristics of intramolecular acyl units (Table 6) [55,57]. Due to increased volume and structural complexity of the acylation product molecule, steric hindrance caused by acylation, the twisted acyl moiety, and the reduction of electron inductive effects, the derivative is prevented from reaching the active site of DPPH, which reduces DPPH free radical scavenging activity [53,60,155]. Therefore, the influence of acyl donors on oxidation resistance of acylation products should be fully considered, particularly in applications. Various methods should be used to study the chemical antioxidant potential of the sample, such as DPPH free radical scavenging ability [51,57], ABTS free radical scavenging method [2], ferric reducing antioxidant power (FRAP) [60], and oxygen free radical absorption capacity (ORAC) assays [61,63].

A correlation of antioxidant activity and anthocyanin content has been reported [156]. Therefore, reducing the degradation of anthocyanins in application will also indirectly improve their antioxidant properties. The combination of anthocyanin and maltodextrin/modified maize starch protects anthocyanin from the damage of oxygen and temperature [79]. The antioxidant activity of sour cherry pomace extract encapsulated in whey and soy proteins improved during the storage period of 4 months [157].

#### 5.2.2. Cellular Antioxidant

In vivo antioxidant assays (cellular antioxidant activity) are a superior approach to investigate the medicinal potential of modified anthocyanins [61]. Cellular antioxidant activity includes cellular adsorption, metabolism, and intracellular distribution of antioxidants [158]. The research by Zhang et al. [61] suggested that acylation with fatty acids improved the cellular uptake of anthocyanins, and the highest intracellular antioxidant activity was achieved with medium-chain C3G-laurate. Moreover, another study found that acylation of cyanindin-3-*O*-glucoside could effectively prevent the release of reactive oxygen species (ROS) caused by oxidative damage and alleviate oxidative stress damage [63]. However, in numerous studies, antioxidant properties of anthocyanins and modified anthocyanins have only been analyzed by simple experimental systems in vitro. Meanwhile, cellular antioxidant activity needs to be paid more attention to improve the application value of anthocyanins in functional foods and medicines.

**Table 6:** Antioxidant properties of anthocyanins and modified anthocyanins.

Anthocyanins	Modified Method	Improvement Effect	Reference
cyanidin-3-*O*-glucoside	co-pigmentation	The DPPH clearance ratio of C3G itself was 83.25 ± 16.50%, and the ratio of C3G in nanocomposites was 87.47 ± 6.69%.	[19]
black rice anthocyanin extracts	double emulsion	The scavenging activities of ABTS radical cation and DPPH radical of all microcapsules ranged from 0.7 to 5.8 μg Trolox/100 g dw and 0.6–3.5 μg Trolox/100 g dw, respectively. The co-pigment addition increased scavenging activities of ABTS radical cation and DPPH radical.	[21]
cyanidin-3-*O*-glucoside	microencapsulation	After intestinal digestion, the ORAC value of anthocyanins in the vesicles was 2.8 times higher than that of free anthocyanins.	[22]
blueberry anthocyanins extracts	enzymatic acylation	The DPPH radical scavenge rate of anthocyanins extracts was 64.75% and increased by 6.56% and 15.21% after grafting with p-coumaric acid and caffeic acid, respectively. Additionally, the inhibition ratio in the β-carotene bleaching assay of the anthocyanins of anthocyanins extracts was 77.11% and increased by 7.93% and 16.86% respectively.	[43]
blackcurrant anthocyanins extracts	enzymatic acylation	The inhibition capacities of acylated products of delphinidin-3-*O*-rutinoside, cyanidin-3-*O*-glucoside, and cyanidin-3-*O*-rutinoside reached 67%, 88%, and 72% of the ability of BHT, respectively, which was significantly higher than unacylated products.	[52]
anthocyanin extracts (cyanidin-3-glucoside	enzymatic acylation	Cyanidin-3-(6″-dihydroferuloyl) glucoside and cyanidin-3-(6″-dihydrosinapoyl) glucoside exhibited better antioxidant activity than cyanidin-3-glucoside.	[57]
raspberry anthocyanin	enzymatic acylation	The acylated anthocyanins effectively prevented the release of ROS caused by oxidative damage and alleviated oxidative stress damage.	[63]
Iranian borage anthocyanins extracts	microencapsulation	In comparison with crude Iranian borage extract, the IC_50_ of microcapsules had a significant decrease at 40 °C during 60 days of storage, and the antioxidant property increased 7.54 times for microcapsules.	[79]
anthocyanin extracts	emulsion	The DPPH radical scavenging potential of anthocyanins encapsulation by the emulsion method (EC50 7.43 mg mL^−1^) was comparatively higher than that of unencapsulation anthocyanins.	[105]
anthocyanin extracts	nanoliposomes as delivery system	Compared with unencapsulated anthocyanins, the anthocyanins in nanoliposomes were more stable and exhibited higher antioxidant activity within 28 days.	[112]
concentrated anthocyanin extract	co-pigmentation	The co-pigmentation of anthocyanin and rutin showed a beneficial effect on antioxidant capacity from the 5 weeks of storage.	[120]
elderberry anthocyanin extracts	microencapsulation	The combination of polysaccharide encapsulation and EGCG copolymerization improved the stability of anthocyanins against high temperature and the presence of ascorbic acid.	[159]

### 5.3. Lipophilicity of Modified Anthocyanins

Since anthocyanins are widely distributed water-soluble pigments in nature, their incorporation into lipid-rich matrices (such as many foods and formulas) is limited. Improvement in the lipophilicity of anthocyanins is mainly accomplished by chemical modification. Essentially, lipophilicity consists of the esterification of a lipophilic moiety (fatty acid or fatty alcohol) on different substrates (phenolic acid, sugar, protein, etc.), which results in new anthocyanin molecules with modified hydrophilic and lipophilic balance [160]. The enhanced lipophilic properties of an acylated derivative will contribute to penetrate into lipid matrices or lipophilic media and expand the scope of application of anthocyanins as colorants from aqueous to fat-rich food matrices [53]. 

Grajeda-Iglesias et al. [161] used octanoyl chloride as an acyl donor to successfully lipophilize anthocyanins at room temperature, significantly improving the lipophilicity of anthocyanins [52]. The octanol/water partition coefficient (log P) was usually used to measure the lipophilicity of acylated derivatives. After acylation with lauric acid, the log P values of acylated anthocyanin derivatives significantly increased from negative to positive, indicating the characteristic transformation from hydrophilicity to lipophilicity [52,162]. Cruz et al. [51] also found that the lipophilicity of anthocyanins is related to the length of the fatty acid chain. 

### 5.4. Bioavailability of Modified Anthocyanins

The bioavailability of anthocyanins is closely related to human health [163]. However, the bioavailability of anthocyanins is typically less than 0.1%, requiring a large amount of administration [7,37,164]. The modification method to improve the bioavailability of anthocyanins can be summarized as: (1) the structure of chemically modified anthocyanins enhances its lipophilicity, improves its ability to freely pass through the gastrointestinal membranes, and increases metabolic efficiency [45,165]; and (2) physical embedding of anthocyanins prevents contact with the protein in the stomach environment and prevents degradation caused by pH changes, thereby allowing smooth release in the intestine and participation in microbial metabolism and blood circulation [35,166]. 

The enhanced lipophilicity of anthocyanins may lead to their improved incorporation into the lipid bilayer of the cell membrane, resulting in better bioavailability in the body as well as greater potential in drug delivery based on liposomes [165]. However, in the stomach environment of pH 1–2, anthocyanins exist as polar flavylium cations, which impedes their passive diffusion through the gastric mucosa [37]. Acylation of anthocyanins could significantly enhance their lipophilicity [53,165], the affinity of the cell membrane, and its ability to freely pass through the gastric mucosa [167]. Additionally, encapsulating anthocyanins through the interaction between wall materials (protein and polysaccharide) and anthocyanins could provide resistance to the effects of digestive enzymes and pH changes in the gastrointestinal tract, which could degrade anthocyanins [94,168]. The low pH of the stomach can easily cause denaturation of protein. Compared with the protein-based wall material, polysaccharide-based wall material has a more significant protective effect on anthocyanins in the stomach [109].

## 6. Physiological Functions of Modified Anthocyanins

Anthocyanins play a significant role in the treatment of cancer [169], inflammation [170,171], neurological diseases, cardiovascular diseases [17,19], etc. and offer multiple benefits for human health. Low absorption stability in the human body and low solubility are significant obstacles in drug delivery of anthocyanins [164]. Most importantly, low permeability of anthocyanins in epithelial cells as well as untargeted release of cancer and inflammatory factors reduce their physiological functions [172]. Therefore, novel and suitable delivery systems are needed to enhance the absorption of anthocyanins in epithelial cells and provide a targeted release to the tumor cells of the anthocyanins [173].

Recently, incorporation of anthocyanin molecules into various carriers was shown to enhance the absorption of anthocyanins in epithelial cells and provide a targeted release to cancer cells, which inevitably increases their anti-cancer activity [174]. On the one hand, no specific receptors on the surface of small intestinal epithelial cells have been found to carry anthocyanins into cells [172]. The mechanism for anthocyanin transport across the epithelium was primarily based on passive diffusion. Nanoparticles enhance absorption of anthocyanins in epithelial cells via endocytosis, enhancing absorption of anthocyanins encapsulated in the gastrointestinal tract [103]. Anthocyanin–fucoidan nanocomplexes are absorbed through endocytosis in the small intestine and have higher cell permeability, absorption, and plasma chemical stability than free anthocyanins [164]. On the other hand, nanocarriers are capable of improving targeting and delivery of polyphenols to cancer cells due to their ability to overcome environmental barriers. Anthocyanin/chitosan (CH)/chondroitin sulfate (CS) nanoparticles induced higher cancer cell apoptosis due to their protective effect of biopolymer particles, which avoided the degradation of anthocyanin and increased the biological activities at the same concentration [175]. Because tumor regions have unique environmental characteristics such as low pH, pH-sensitive polymeric anthocyanin carriers have been designated as promising candidates for efficient tumor therapy [176,177,178]. The pH-responsive drug-delivery system of black carrot anthocyanins loaded in halloysite nanotubes achieves targeted release of cancer cells [38]. As compared to anthocyanins, the viability of both breast cancer and colon cancer cell lines was reduced by two-fold against anthocyanin-loaded HNT. 

## 7. Application Challenges of Anthocyanins

While considerable research has been carried out regarding the modification of anthocyanins, there are still a series of problems in practical applications, especially the safety of modified products. For instance, the safety of the product is difficult to predict due to the introduction of hazardous residuals in the chemical modification. The organic solvents that are dedicated to acylation are harmful to human health, such as tert-butanol, acetone, acetonitrile, etc. [46,179]. The crash of encapsulation particles into the shell seems to be a potential hazard. The high temperature during processing leads to the denaturation of wall materials such as proteins or reacts with carbonyl compounds, which may form harmful products, such as Maillard reaction/caramelization products, acrylamide, and so on [81,86]. Therefore, not only do harmful residues introduced by modification need more attention, but the stabilization processing of anthocyanins also needs more studies. 

On the other hand, the high cost and low yield limit the large-scale production of modified anthocyanins. Although the properties of anthocyanin were improved by acylation, many factors caused the acylation of anthocyanin to stagnate in the laboratory stage, such as unidentified structures and low conversion rate. The conversion rates of blueberry anthocyanin enzymatic acylation with coumaric acid and caffeic acid are less than 10% [43]. The structures of a considerable number of acylated products have not been analyzed in detail [55,180]. The drying technology used in microencapsulation increases the cost; this is true for both freeze-drying technology that uses vacuum technology or spray-drying technology that is prone to waste materials and loss of fine particles in the exhaust gas [181]. The stability and solubility of modified anthocyanins only were studied in the model solution, and their properties in complex food systems are still unclear [64,182]. 

Additionally, the classical microencapsulation methods can significantly improve the stability of anthocyanins but, in general, can deliver the bio-accessible and/or bioavailable anthocyanins to their absorption sites [7]. It is unknown whether acylated anthocyanin affects the production and efficiency of its metabolites [144]. Therefore, there is also a concern about the bioavailability of modified anthocyanins. Increased in vitro stability and bioavailability of modified anthocyanins, such as stabilized anthocyanins, require more attention to target absorption and metabolism pathways [64].

Overall, it is necessary to conduct further scientific and systemic research on the stability, bioavailability, toxicity, and metabolism of modified anthocyanin. Strict assessments can accelerate the application of anthocyanins in the food industry. Furthermore, the combination of the best performance of the product with environmental protection, high yield, and low cost should also be considered.

## 8. Conclusions

The modification of anthocyanins has gradually become an effective measure to overcome the instability of anthocyanins, which leads to low bioavailability and physiological function obstacles. This review not only focused on the advanced modification strategies but also summarized the effects of modification technologies on the antioxidant capacity, lipophilicity, and bioavailability of anthocyanins. Modification (e.g., co-pigmentation, acylation, microencapsulation, and pickering emulsion) has been reported to be an effective method for maintaining and/or improving the shelf-life and stability of anthocyanins due to controlling the degradation of anthocyanins during storage and gastrointestinal digestion. 

The improved stability of modified anthocyanins significantly improves their bioavailability and further promotes their physiological functions. In addition, the current challenges and technical limitations in stabilizing anthocyanins were also identified by us. This includes how the introduction of organic reagents in the acylation process threatens product safety and how overcoming the high cost of microencapsulation requires technological innovation. The strategies of high yield and low cost and improving the stability of anthocyanin deserve more attention in the field of food additives, food colorants, and smart packaging indicator materials. In the fields of dietary supplement and disease prevention, we should fully understand and clarify the mechanisms of absorption and metabolism of anthocyanins in the human body.

## Figures and Tables

**Table 2:** Enzymatic acylation conditions and acylation rate of anthocyanins.

Products	Acyl Donor	Enzyme	Acylation Rate		Reaction Medium	Reference
laurylmonoesters of cyanidin-3,5-*O*-diglucoside	lauric acid	lipase Fermase CALB™ 10,000	Only acetone showed synthesis of anthocyanin fatty acid esters and the conversion rate reached 63%		acetone	[2]
					t-butanol	
					t-amyl alcohol	
cyanidin 3-(6′′-benzoyl)-glucoside; cyaniding-3-(6′′-salicyloyl)- glucoside and cyanidin 3-(6′′-cinnamoyl)-glucoside	aromatic acid methyl esters	Candida antarctica lipase B	90.92%		pyridine	[13]
anthocyanins (Co-An)	p-coumaric acid	Lipase: Novozym 435	Acylation degrees 5.38%		tetrahydrofuran (THF)	[43]
anthocyanins (Ca-An)	caffffeic acid		Acylation degrees 5.68%			
-	caprylic acid	Candida antarctica lipase B	40%		anhydrous 2-methyl-2-butanol	[51]
cyanidin-3-*O*-(6′′-dodecanoyl) galactoside	lauric acid	Novezym 435	The conversion rate of tert-butanol reach by 73%		acetone	[52]
					acetonitrile	
					tert-butanol	
					tert-amyl alcohol	
delphinidin-3-glucoside-6′′-*O*-octanoate and cyanidin-3-glucoside-6′′-*O*-octanoate	octanoic acid	Candida antarctica lipase B	-		dry acetonitrile:DMSO 10:1 (*v*/*v*)	[57]
			-			
cyanidin-3,5-diglycoside cinnamic acid vinyl ester acylate	vinyl cinnamate	Candida antarctica lipase B	85.7%		dry pyridine	[57]
					tert-butanol	
					2-methyl-2-butanol	
	lauric acid	Candida antarctica lipase B	Ethanol with a volume fraction of 10%	Ethanol with a volume fraction of 20%	anhydrous 2-methyl-2-butanol	[59]
Delphidin-3-*O*-glucoside lauric acid acylate			77	85		
Delphidin-3-*O*-rutinoside lauric acid acylate			72	74		
Cyanidin-3-*O*-glucoside lauric acid acylate			66	88		
Cyanidin-3-*O*-rutinoside lauric acid acylate			62	63		
cyanidin-3-glucoside-fatty acid conjugate	octanoic acid	Candida antarctica lipase B	-		2-methyl-2-butanol	[59]
	octadecenoic acid	Candida antarctica lipase B	21.2%		anhydrous 2-methyl-2-butanol	[60]
cyanidin-3-(6′′-n-octanoyl)-glucoside, cyanidin-3-(6′′-lauroyl)-glucoside, and cyanidin-3-(6′′-myristoyl)-glucoside	fatty acid methyl esters	Lipozyme 435	94%		tertamyl alcohol	[61]
cyanidin-3-glucoside-octanoic acid acylate	octanoic acid	Novozymes 435	47.1%		tertiary butanol	[62]

## Data Availability

The data used to support the findings of this study can be made available by the corresponding author upon request.

## References

[1] Cavalcanti R.N., Santos D.T., Meireles M. (2011). Non-thermal stabilization mechanisms of anthocyanins in model and food systems—An overview. Food Res. Int..

[2] Marathe S.J., Shah N.N., Bajaj S.R., Singhal R.S. (2021). Esterification of anthocyanins isolated from floral waste: Characterization of the esters and their application in various food systems. Food Biosci..

[3] Qin Y., Yun D., Xu F., Chen D., Kan J., Liu J. (2021). Smart packaging films based on starch/polyvinyl alcohol and Lycium ruthenicum anthocyanins-loaded nano-complexes: Functionality, stability and application. Food Hydrocoll..

[4] Chorfa N., Savard S., Belkacemi K. (2016). An efficient method for high-purity anthocyanin isomers isolation from wild blueberries and their radical scavenging activity. Food Chem..

[5] EU Commission (2012). Commission regulation (EU) no 231/2012 of 9 March 2012 laying down specifications for food additives listed in annexes II and III to regulation (EC) no 1333/2008 of the European Parliament and of the Council. Off. J. Eur. Communities.

[6] Kowalska G., Wyrostek J., Kowalski R., Pankiewicz U. (2021). Evaluation of glycerol usage for the extraction of anthocyanins from black chokeberry and elderberry fruits. J. Appl. Res. Med. Aromat. Plants.

[7] Tarone A.G., Cazarin C.B.B., Marostica M.R. (2020). Anthocyanins: New techniques and challenges in microencapsulation. Food Res. Int..

[8] Xu X.J., Fang S., Li Y.H., Zhang F., Shao Z.P., Zeng Y.T., Chen J., Meng Y.C. (2019). Effects of low acyl and high acyl gellan gum on the thermal stability of purple sweet potato anthocyanins in the presence of ascorbic acid. Food Hydrocoll..

[9] Peng F.A., Fz A., Sz A., Qc A., Yh A., Jie C.B. (2020). Acylation of blueberry anthocyanins with maleic acid: Improvement of the stability and its application potential in intelligent color indicator packing materials. Dye. Pigment..

[10] Kong J.M., Chia L.S., Goh N.K., Chia T.F., Brouillard R. (2003). Analysis and biological activities of anthocyanins. Phytochemistry.

[11] Oliveira H., Perez-Gregorio R., de Freitas V., Mateus N., Fernandes I. (2019). Comparison of the in vitro gastrointestinal bioavailability of acylated and non-acylated anthocyanins: Purple-fleshed sweet potato vs red wine. Food Chem..

[12] Gowd V., Jia Z., Chen W. (2017). Anthocyanins as promising molecules and dietary bioactive components against diabetes- A review of recent advances. Trends Food Sci. Technol..

[13] Yan Z., Li C., Zhang L., Liu Q., Ou S., Zeng X. (2016). Enzymatic Acylation of Anthocyanin Isolated from Black Rice with Methyl Aromatic Acid Ester as Donor: Stability of the Acylated Derivatives. J. Agric. Food Chem..

[14] Wang Z., Sun L., Fang Z., Nisar T., Zou L., Li D., Guo Y. (2021). Lycium ruthenicum Murray anthocyanins effectively inhibit α-glucosidase activity and alleviate insulin resistance. Food Biosci..

[15] Yu W., Gao J., Hao R., Zhang C., Liu H., Fan J., Wei J. (2021). *Aronia melanocarpa* Elliot anthocyanins inhibit colon cancer by regulating glutamine metabolism. Food Biosci..

[16] Jokioja J., Linderborg K.M., Kortesniemi M., Nuora A., Heinonen J., Sainio T., Viitanen M., Kallio H., Yang B. (2020). Anthocyanin-rich extract from purple potatoes decreases postprandial glycemic response and affects inflammation markers in healthy men. Food Chem..

[17] Kruger M.J., Davies N., Myburgh K.H., Lecour S. (2014). Proanthocyanidins, anthocyanins and cardiovascular diseases. Food Res. Int..

[18] Garcia C., Blesso C.N. (2021). Antioxidant properties of anthocyanins and their mechanism of action in atherosclerosis. Free Radic. Biol. Med..

[19] Li P., Feng D., Yang D., Li X., Sun J., Wang G., Tian L., Jiang X., Bai W. (2021). Protective effects of anthocyanins on neurodegenerative diseases. Trends Food Sci. Technol..

[20] Sw A., Xy A., Yue S.A., Jin L.A., Py B., Xg A. (2021). Nanocomplexes derived from chitosan and whey protein isolate enhance the thermal stability and slow the release of anthocyanins in simulated digestion and prepared instant coffee. Food Chem..

[21] Kanha N., Surawang S., Pitchakarn P., Laokuldilok T. (2020). Microencapsulation of copigmented anthocyanins using double emulsion followed by complex coacervation: Preparation, characterization and stability. LWT.

[22] Fan L., Chen Q., Mairiyangu Y., Wang Y., Liu X. (2021). Stable vesicle self-assembled from phospholipid and mannosylerythritol lipid and its application in encapsulating anthocyanins. Food Chem..

[23] Yong H., Liu J. (2020). Recent advances in the preparation, physical and functional properties, and applications of anthocyanins-based active and intelligent packaging films. Food Packag. Shelf Life.

[24] Yong H., Wang X., Bai R., Miao Z., Liu J. (2018). Development of antioxidant and intelligent pH-sensing packaging films by incorporating purple-fleshed sweet potato extract into chitosan matrix. Food Hydrocoll..

[25] De Aguiar Cipriano P., Ekici L., Barnes R.C., Gomes C., Talcott S.T. (2015). Pre-heating and polyphenol oxidase inhibition impact on extraction of purple sweet potato anthocyanins. Food Chem..

[26] Oliveira J., Bras N.F., da Silva M.A., Mateus N., Parola A.J., de Freitas V. (2014). Grape anthocyanin oligomerization: A putative mechanism for red color stabilization?. Phytochemistry.

[27] Chandra Singh M., Kelso C., Price W.E., Probst Y. (2020). Validated liquid chromatography separation methods for identification and quantification of anthocyanins in fruit and vegetables: A systematic review. Food Res. Int..

[28] Betz M., Kulozik U. (2011). Microencapsulation of bioactive bilberry anthocyanins by means of whey protein gels. Procedia Food Sci..

[29] Chen J.Y., Du J., Li M.L., Li C.M. (2020). Degradation kinetics and pathways of red raspberry anthocyanins in model and juice systems and their correlation with color and antioxidant changes during storage. LWT.

[30] Verbeyst L., Oey I., Van der Plancken I., Hendrickx M., Van Loey A. (2010). Kinetic study on the thermal and pressure degradation of anthocyanins in strawberries. Food Chem..

[31] Attaribo T., Jiang X., Huang G., Zhang B., Xin X., Zhang Y., Zhang N., Gui Z. (2020). Studies on the interactional characterization of preheated silkworm pupae protein (SPP) with anthocyanins (C3G) and their effect on anthocyanin stability. Food Chem..

[32] Amr A., Al-Tamimi E. (2007). Stability of the crude extracts of Ranunculus asiaticus anthocyanins and their use as food colourants. Int. J. Food Sci. Technol..

[33] Mullen W., Edwards C.A., Crozier A. (2006). Absorption, excretion and metabolite profiling of methyl-, glucuronyl-, glucosyl- and sulpho-conjugates of quercetin in human plasma and urine after ingestion of onions. Br. J. Nutr..

[34] Alvarez-Suarez J.M., Cuadrado C., Redondo I.B., Giampieri F., González-Paramás A.M., Santos-Buelga C. (2021). Novel approaches in anthocyanin research—Plant fortification and bioavailability issues. Trends Food Sci. Technol..

[35] Chi J., Ge J., Yue X., Liang J., Sun Y., Gao X., Yue P. (2019). Preparation of nanoliposomal carriers to improve the stability of anthocyanins. LWT.

[36] Salah M., Mansour M., Zogona D., Xu X. (2020). Nanoencapsulation of anthocyanins-loaded beta-lactoglobulin nanoparticles: Characterization, stability, and bioavailability in vitro. Food Res. Int..

[37] Mueller D., Jung K., Winter M., Rogoll D., Melcher R., Kulozik U., Schwarz K., Richling E. (2018). Encapsulation of anthocyanins from bilberries—Effects on bioavailability and intestinal accessibility in humans. Food Chem..

[38] Kamonpatana K., Failla M.L., Kumar P.S., Giusti M.M. (2014). Anthocyanin structure determines susceptibility to microbial degradation and bioavailability to the buccal mucosa. J. Agric. Food Chem..

[39] Talavera S., Felgines C., Texier O., Besson C., Lamaison J.L., Remesy C. (2003). Anthocyanins are efficiently absorbed from the stomach in anesthetized rats. J. Nutr..

[40] Fernandes I., Faria A., Calhau C., de Freitas V., Mateus N. (2014). Bioavailability of anthocyanins and derivatives. J. Funct. Foods.

[41] Han F., Oliveira H., Bras N.F., Fernandes I., Cruz L., De Freitas V., Mateus N. (2020). In vitro gastrointestinal absorption of red wine anthocyanins—Impact of structural complexity and phase II metabolization. Food Chem..

[42] Oliveira H., Basilio N., Pina F., Fernandes I., de Freitas V., Mateus N. (2019). Purple-fleshed sweet potato acylated anthocyanins: Equilibrium network and photophysical properties. Food Chem..

[43] Liu J., Zhuang Y., Hu Y., Xue S., Li H., Chen L., Fei P. (2020). Improving the color stability and antioxidation activity of blueberry anthocyanins by enzymatic acylation with p-coumaric acid and caffeic acid. LWT.

[44] Zhao C.L., Yu Y.Q., Chen Z.J., Wen G.S., Wei F.G., Zheng Q., Wang C.D., Xiao X.L. (2017). Stability-increasing effects of anthocyanin glycosyl acylation. Food Chem..

[45] Giusti M.M., Wrolstad R.E. (2003). Acylated anthocyanins from edible sources and their applications in food systems. Biochem. Eng. J..

[46] Stevenson D.E., Wibisono R., Jensen D.J., Stanley R.A., Cooney J.M. (2006). Direct acylation of flavonoid glycosides with phenolic acids catalysed by Candida antarctica lipase B (Novozym 435^®^). Enzym. Microb. Technol..

[47] Fenger J.A., Roux H., Robbins R.J., Collins T.M., Dangles O. (2021). The influence of phenolic acyl groups on the color of purple sweet potato anthocyanins and their metal complexes. Dye. Pigment..

[48] Moloney M., Robbins R.J., Collins T.M., Kondo T., Yoshida K., Dangles O. (2018). Red cabbage anthocyanins: The influence of d-glucose acylation by hydroxycinnamic acids on their structural transformations in acidic to mildly alkaline conditions and on the resulting color. Dye. Pigment..

[49] Cruz L., Fernandes V.C., Araújo P., Mateus N., de Freitas V. (2015). Synthesis, characterisation and antioxidant features of procyanidin B4 and malvidin-3-glucoside stearic acid derivatives. Food Chem..

[50] Xiao D., Jin X., Song Y., Zhang Y., Li X., Wang F. (2021). Enzymatic acylation of proanthocyanidin dimers from Acacia mearnsii bark: Effect on lipophilic and antioxidant properties. J. Bioresour. Bioprod..

[51] Cruz L., Guimaraes M., Araujo P., Evora A., de Freitas V., Mateus N. (2017). Malvidin 3-Glucoside-Fatty Acid Conjugates: From Hydrophilic toward Novel Lipophilic Derivatives. J. Agric. Food Chem..

[52] Yang W., Kortesniemi M., Ma X., Zheng J., Yang B. (2019). Enzymatic acylation of blackcurrant (*Ribes nigrum*) anthocyanins and evaluation of lipophilic properties and antioxidant capacity of derivatives. Food Chem..

[53] Yang W., Kortesniemi M., Yang B., Zheng J. (2018). Enzymatic Acylation of Anthocyanins Isolated from Alpine Bearberry (*Arctostaphylos alpina*) and Lipophilic Properties, Thermostability, and Antioxidant Capacity of the Derivatives. J. Agric. Food Chem..

[54] Mazuco R.A., Cardoso P.M.M., Bindaco E.S., Scherer R., Castilho R.O., Faraco A.A.G., Ruas F.G., Oliveira J.P., Guimaraes M.C.C., de Andrade T.U. (2018). Maltodextrin and Gum Arabic-Based Microencapsulation Methods for Anthocyanin Preservation in Jucara Palm (*Euterpe edulis Martius*) Fruit Pulp. Plant Foods Hum. Nutr..

[55] Matsufuji H., Kido H., Misawa H., Yaguchi J., Otsuki T., Chino M., Takeda M., Yamagata K. (2007). Stability to light, heat, and hydrogen peroxide at different pH values and DPPH radical scavenging activity of acylated anthocyanins from red radish extract. J. Agric. Food Chem..

[56] Hazarika S., Goswami P., Dutta N.N. (2003). Lipase catalysed transesterification of 2-o-benzylglycerol with vinyl acetate: Solvent effect. Chem. Eng. J..

[57] Fernandez-Aulis F., Torres A., Sanchez-Mendoza E., Cruz L., Navarro-Ocana A. (2020). New acylated cyanidin glycosides extracted from underutilized potential sources: Enzymatic synthesis, antioxidant activity and thermostability. Food Chem..

[58] Cai X., Du X., Cui D., Wang X., Yang Z., Zhu G. (2019). Improvement of stability of blueberry anthocyanins by carboxymethyl starch/xanthan gum combinations microencapsulation. Food Hydrocoll..

[59] Marquez-Rodriguez A.S., Guimaraes M., Mateus N., de Freitas V., Ballinas-Casarrubias M.L., Fuentes-Montero M.E., Salas E., Cruz L. (2021). Disaccharide anthocyanin delphinidin 3-*O*-sambubioside from *Hibiscus sabdariffa* L.: Candida antarctica lipase B-catalyzed fatty acid acylation and study of its color properties. Food Chem..

[60] Cruz L., Fernandes I., Guimaraes M., de Freitas V., Mateus N. (2016). Enzymatic synthesis, structural characterization and antioxidant capacity assessment of a new lipophilic malvidin-3-glucoside-oleic acid conjugate. Food Funct..

[61] Zhang P., Liu S., Zhao Z., You L., Harrison M.D., Zhang Z. (2021). Enzymatic acylation of cyanidin-3-glucoside with fatty acid methyl esters improves stability and antioxidant activity. Food Chem..

[62] Lin Y., Li C., Shao P., Jiang L., Chen B., Farag M.A. (2022). Enzymatic acylation of cyanidin-3-*O*-glucoside in raspberry anthocyanins for intelligent packaging: Improvement of stability, lipophilicity and functional properties. Curr. Res. Nutr. Food Sci..

[63] Teng H., Mi Y., Cao H., Chen L. (2022). Enzymatic acylation of raspberry anthocyanin: Evaluations on its stability and oxidative stress prevention. Food Chem..

[64] Cai D., Li X., Chen J., Jiang X., Ma X., Sun J., Tian L., Vidyarthi S.K., Xu J., Pan Z. (2022). A comprehensive review on innovative and advanced stabilization approaches of anthocyanin by modifying structure and controlling environmental factors. Food Chem..

[65] Kontogianni A., Skouridou V., Sereti V., Stamatis H., Kolisis F.N. (2001). Regioselective acylation of flavonoids catalyzed by lipase in low toxicity media. Eur. J. Lipid Sci. Technol..

[66] Bakker J., Timberlake C.F. (1997). Isolation, Identification, and Characterization of New Color-Stable Anthocyanins Occurring in Some Red Wines. J. Agric. Food Chem..

[67] Rentzsch M., Schwarz M., Winterhalter P. (2007). Pyranoanthocyanins—An overview on structures, occurrence, and pathways of formation. Trends Food Sci. Technol..

[68] Liu S., Laaksonen O., Yang W., Zhang B., Yang B. (2020). Pyranoanthocyanins in bilberry (*Vaccinium myrtillus* L.) wines fermented with Schizo saccharomyces pombe and their evolution during aging. Food Chem..

[69] He J., Carvalho A.R., Mateus N., De Freitas V. (2010). Spectral Features and Stability of Oligomeric Pyranoanthocyanin-flavanol Pigments Isolated from Red Wines. J. Agric. Food Chem..

[70] Božič J.T., Butinar L., Albreht A., Vovk I., Korte D., Vodopivec B.M. (2020). The impact of Saccharomyces and non-Saccharomyces yeasts on wine colour: A laboratory study of vinylphenolic pyranoanthocyanin formation and anthocyanin cell wall adsorption. LWT.

[71] Quaglieri C., Jourdes M., Waffo-Teguo P., Teissedre P.-L. (2017). Updated knowledge about pyranoanthocyanins: Impact of oxygen on their contents, and contribution in the winemaking process to overall wine color. Trends Food Sci. Technol..

[72] Arroyo-Maya I.J., McClements D.J. (2015). Biopolymer nanoparticles as potential delivery systems for anthocyanins: Fabrication and properties. Food Res. Int..

[73] Zeng Y.J., Xu P., Yang H.R., Zong M.H., Lou W.Y. (2018). Purification of anthocyanins from saskatoon berries and their microencapsulation in deep eutectic solvents. LWT.

[74] Norkaew O., Thitisut P., Mahatheeranont S., Pawin B., Sookwong P., Yodpitak S., Lungkaphin A. (2019). Effect of wall materials on some physicochemical properties and release characteristics of encapsulated black rice anthocyanin microcapsules. Food Chem..

[75] Balakrishnan M., Gayathiri S., Preetha P., Pandiselvam R., Jeevarathinam G., Delfiya D.S.A., Kothakota A. (2021). Microencapsulation of bixin pigment by spray drying: Evaluation of characteristics. LWT.

[76] Righi da Rosa J., Cezimbra Weis G.C., Bolson Moro K.I., Sasso Robalo S., Elias Assmann C., Picolli da Silva L., Irineu Muller E., de Bona da Silva C., Ragagnin de Menezes C., Severo da Rosa C. (2021). Effect of wall materials and storage temperature on anthocyanin stability of microencapsulated blueberry extract. LWT.

[77] Akhavan Mahdavi S., Jafari S.M., Assadpoor E., Dehnad D. (2016). Microencapsulation optimization of natural anthocyanins with maltodextrin, gum Arabic and gelatin. Int. J. Biol. Macromol..

[78] Aksoylu Özbek Z., Günç Ergönül P. (2020). Optimisation of wall material composition of freeze–dried pumpkin seed oil microcapsules: Interaction effects of whey protein, maltodextrin, and gum Arabic by D–optimal mixture design approach. Food Hydrocoll..

[79] Mehran M., Masoum S., Memarzadeh M. (2020). Improvement of thermal stability and antioxidant activity of anthocyanins of Echium amoenum petal using maltodextrin/modified starch combination as wall material. Int. J. Biol. Macromol..

[80] Oancea A.M., Hasan M., Vasile A.M., Barbu V., Enachi E., Bahrim G., Rapeanu G., Silvi S., Stănciuc N. (2018). Functional evaluation of microencapsulated anthocyanins from sour cherries skins extract in whey proteins isolate. LWT.

[81] Mansour M., Salah M., Xu X. (2020). Effect of microencapsulation using soy protein isolate and gum arabic as wall material on red raspberry anthocyanin stability, characterization, and simulated gastrointestinal conditions. Ultrason. Sonochem..

[82] Kuck L.S., Wesolowski J.L., Norena C.P.Z. (2017). Effect of temperature and relative humidity on stability following simulated gastro-intestinal digestion of microcapsules of Bordo grape skin phenolic extract produced with different carrier agents. Food Chem..

[83] Ersus S., Yur Da Gel U. (2007). Microencapsulation of anthocyanin pigments of black carrot (*Daucus carota* L.) by spray drier. J. Food Eng..

[84] Sakulnarmrat K., Wongsrikaew D., Konczak I. (2021). Microencapsulation of red cabbage anthocyanin-rich extract by drum drying technique. LWT.

[85] Wang Y., Lu Z., Lv F., Bie X. (2009). Study on microencapsulation of curcumin pigments by spray drying. Eur. Food Res. Technol..

[86] Pereira Souza A.C., Deyse Gurak P., Damasceno Ferreira Marczak L. (2017). Maltodextrin, pectin and soy protein isolate as carrier agents in the encapsulation of anthocyanins-rich extract from jaboticaba pomace. Food Bioprod. Process..

[87] Fredes C., Becerra C., Parada J., Robert P. (2018). The Microencapsulation of Maqui (*Aristotelia chilensis* (Mol.) Stuntz) Juice by Spray-Drying and Freeze-Drying Produces Powders with Similar Anthocyanin Stability and Bioaccessibility. Molecules.

[88] Enache I.M., Vasile A.M., Enachi E., Barbu V., Stănciuc N., Vizireanu C. (2020). Co-microencapsulation of anthocyanins from cornelian cherry fruits and lactic acid bacteria in biopolymeric matrices by freeze-drying: Evidences on functional properties and applications in food. Polymers.

[89] Constantin O.E., Stănciuc N., Yan Y., Ghinea I.O., Ungureanu C., Cîrciumaru A., Wang D., Ulrih N.P., Râpeanu G. (2021). Polymers and protein-associated vesicles for the microencapsulation of anthocyanins from grape skins used for food applications. J. Sci. Food Agric..

[90] Kanokpanont S., Yamdech R., Aramwit P. (2018). Stability enhancement of mulberry-extracted anthocyanin using alginate/chitosan microencapsulation for food supplement application. Artif. Cells Nanomed. Biotechnol..

[91] Machado M.H., da Rosa Almeida A., Maciel M.V.D.O.B., Vitorino V.B., Bazzo G.C., Rosa C.G., Barreto P.L.M. (2022). Microencapsulation by spray drying of red cabbage anthocyanin-rich extract for the production of a natural food colorant. Biocatal. Agric. Biotechnol..

[92] Daniele D., Afonso M.R., Eduardo B., Eliane M., Frederico A., Márcia C., Nataly D., Carolina T. (2018). Increased thermal stability of anthocyanins at pH 4.0 by guar gum in aqueous dispersions and in double emulsions W/O/W. Int. J. Biol. Macromol..

[93] Rui Z.A., Lan Z.A., Jia L.A., Ho B., Ning Y.A., Wj A., Zz A., Sl A., Jh A. (2021). Microencapsulation of anthocyanins extracted from grape skin by emulsification/internal gelation followed by spray/freeze-drying techniques: Characterization, stability and bioaccessibility. LWT.

[94] Wang W., Jung J., Zhao Y. (2017). Chitosan-cellulose nanocrystal microencapsulation to improve encapsulation efficiency and stability of entrapped fruit anthocyanins. Carbohydr. Polym..

[95] Gonzalez Ortiz D., Pochat-Bohatier C., Cambedouzou J., Bechelany M., Miele P. (2020). Current trends in pickering emulsions: Particle morphology and applications. Engineering.

[96] Xia T., Xue C., Wei Z. (2021). Physicochemical characteristics, applications and research trends of edible pickering emulsions. Trends Food Sci. Technol..

[97] Cui F., Zhao S., Guan X., McClements D.J., Liu X., Liu F., Ngai T. (2021). Polysaccharide-based pickering emulsions: Formation, stabilization and applications. Food Hydrocoll..

[98] Yang J., Gu Z., Cheng L., Li Z., Li C., Ban X., Hong Y. (2021). Preparation and stability mechanisms of double emulsions stabilized by gelatinized native starch. Carbohydr. Polym..

[99] Shao P., Feng J., Sun P., Xiang N., Lu B., Qiu D. (2020). Recent advances in improving stability of food emulsion by plant polysaccharides. Food Res. Int..

[100] Chen S., Han Y., Jian L., Liao W., Zhang Y., Gao Y. (2020). Fabrication, characterization, physicochemical stability of zein-chitosan nanocomplex for co-encapsulating curcumin and resveratrol. Carbohydr. Polym..

[101] Han J., Chen F., Gao C., Zhang Y., Tang X. (2020). Environmental stability and curcumin release properties of pickering emulsion stabilized by chitosan/gum arabic nanoparticles. Int. J. Biol. Macromol..

[102] Ge J., Yue P., Chi J., Liang J., Gao X. (2018). Formation and stability of anthocyanins-loaded nanocomplexes prepared with chitosan hydrochloride and carboxymethyl chitosan. Food Hydrocoll..

[103] Zhao X., Zhang X., Tie S., Hou S., Wang H., Song Y., Rai R., Tan M. (2020). Facile synthesis of nano-nanocarriers from chitosan and pectin with improved stability and biocompatibility for anthocyanins delivery: An in vitro and in vivo study. Food Hydrocoll..

[104] Ju M., Zhu G., Huang G., Shen X., Zhang Y., Jiang L., Sui X. (2020). A novel pickering emulsion produced using soy protein-anthocyanin complex nanoparticles. Food Hydrocoll..

[105] Nazareth M.S., Shreelakshmi S.V., Rao P.J., Shetty N.P. (2021). Micro and nanoemulsions of Carissa spinarum fruit polyphenols, enhances anthocyanin stability and anti-quorum sensing activity: Comparison of degradation kinetics. Food Chem..

[106] Sui X., Sun H., Qi B., Zhang M., Li Y., Jiang L. (2018). Functional and conformational changes to soy proteins accompanying anthocyanins: Focus on covalent and non-covalent interactions. Food Chem..

[107] Osvaldt Rosales T.K., Pessoa da Silva M., Lourenço F.R., Hassimotto N.M.A., Fabi J.P. (2021). Nanoencapsulation of anthocyanins from blackberry (*Rubus* spp.) through pectin and lysozyme self-assembling. Food Hydrocoll..

[108] Marefati A., Sjöö M., Timgren A., Dejmek P., Rayner M. (2015). Fabrication of encapsulated oil powders from starch granule stabilized W/O/W pickering emulsions by freeze-drying. Food Hydrocoll..

[109] Lin X., Li S., Yin J., Chang F., Wang C., He X., Huang Q., Zhang B. (2020). Anthocyanin-loaded double pickering emulsion stabilized by octenylsuccinate quinoa starch: Preparation, stability and in vitro gastrointestinal digestion. Int. J. Biol. Macromol..

[110] Liu J., Tan Y., Zhou H., Mundo J.L.M., McClements D.J. (2019). Protection of anthocyanin-rich extract from pH-induced color changes using water-in-oil-in-water emulsions. J. Food Eng..

[111] Huang Y., Zhou W. (2019). Microencapsulation of anthocyanins through two-step emulsification and release characteristics during in vitro digestion. Food Chem..

[112] Sun Y., Chi J., Ye X., Wang S., Gao X. (2020). Nanoliposomes as delivery system for anthocyanins: Physicochemical characterization, cellular uptake, and antioxidant properties. LWT.

[113] Castañeda-Ovando A., Pacheco-Hernández M.d.L., Páez-Hernández M.E., Rodríguez J.A., Galán-Vidal C.A. (2009). Chemical studies of anthocyanins: A review. Food Chem..

[114] Sari P., Wijaya C.H., Sajuthi D., Supratman U. (2012). Colour properties, stability, and free radical scavenging activity of jambolan (*Syzygium cumini*) fruit anthocyanins in a beverage model system: Natural and copigmented anthocyanins. Food Chem..

[115] Huang Y., Zhou S., Zhao G., Ye F. (2021). Destabilisation and stabilisation of anthocyanins in purple-fleshed sweet potatoes: A review. Trends Food Sci. Technol..

[116] Cortez R., Luna-Vital D., Margulis D., Mejia E. (2017). Natural Pigments: Stabilization Methods of Anthocyanins for Food Applications. Compr. Rev. Food Sci. Food Saf..

[117] Zhang X.K., He F., Zhang B., Reeves M.J., Liu Y., Zhao X., Duan C.Q. (2018). The effect of prefermentative addition of gallic acid and ellagic acid on the red wine color, copigmentation and phenolic profiles during wine aging. Food Res. Int..

[118] Molaeafard S., Jamei R., Poursattar Marjani A. (2021). Co-pigmentation of anthocyanins extracted from sour cherry (*Prunus cerasus* L.) with some organic acids: Color intensity, thermal stability, and thermodynamic parameters. Food Chem..

[119] Zhang B., He F., Zhou P.P., Liu Y., Duan C.Q. (2015). Copigmentation between malvidin-3-*O*-glucoside and hydroxycinnamic acids in red wine model solutions: Investigations with experimental and theoretical methods. Food Res. Int..

[120] Hernandez-Herrero J.A., Frutos M.J. (2015). Influence of rutin and ascorbic acid in colour, plum anthocyanins and antioxidant capacity stability in model juices. Food Chem..

[121] Chung C., Rojanasasithara T., Mutilangi W., McClements D.J. (2016). Stabilization of natural colors and nutraceuticals: Inhibition of anthocyanin degradation in model beverages using polyphenols. Food Chem..

[122] Chung C., Rojanasasithara T., Mutilangi W., McClements D.J. (2017). Stability improvement of natural food colors: Impact of amino acid and peptide addition on anthocyanin stability in model beverages. Food Chem..

[123] Li Y., Yao L., Zhang L., Zhang Y., Zheng T., Liu L., Zhang L. (2021). Enhanced physicochemical stabilities of cyanidin-3-*O*-glucoside via combination with silk fibroin peptide. Food Chem..

[124] Condurache N.N., Aprodu I., Grigore-Gurgu L., Petre B.A., Enachi E., Rapeanu G., Bahrim G.E., Stanciuc N. (2020). Fluorescence spectroscopy and molecular modeling of anthocyanins binding to bovine lactoferrin peptides. Food Chem..

[125] Ren S., Giusti M.M. (2021). The effect of whey protein concentration and preheating temperature on the color and stability of purple corn, grape and black carrot anthocyanins in the presence of ascorbic acid. Food Res. Int..

[126] Teixeira N., Cruz L., Bras N.F., Mateus N., Ramos M.J., de Freitas V. (2013). Structural features of copigmentation of oenin with different polyphenol copigments. J. Agric. Food Chem..

[127] Tang P., Giusti M.M. (2020). Metal chelates of petunidin derivatives exhibit enhanced color and stability. Foods.

[128] Shiono M., Matsugaki N., Takeda K. (2005). Structure of the blue cornflower pigment. Nature.

[129] Sasaki N., Nishizaki Y., Ozeki Y., Miyahara T. (2014). The role of acyl-glucose in anthocyanin modifications. Molecules.

[130] Fu Y., Liu W., Soladoye O.P. (2021). Towards innovative food processing of flavonoid compounds: Insights into stability and bioactivity. LWT.

[131] Esfanjani A.F., Assadpour E., Jafari S.M. (2018). Improving the bioavailability of phenolic compounds by loading them within lipid-based nanocarriers. Trends Food Sci. Technol..

[132] McClements D.J. (2020). Advances in nanoparticle and microparticle delivery systems for increasing the dispersibility, stability, and bioactivity of phytochemicals. Biotechnol. Adv..

[133] Klisurova D., Petrova I., Ognyanov M., Georgiev Y., Kratchanova M., Denev P. (2019). Co-pigmentation of black chokeberry (*Aronia melanocarpa*) anthocyanins with phenolic co-pigments and herbal extracts. Food Chem..

[134] Fan L., Wang Y., Xie P., Zhang L., Li Y., Zhou J. (2019). Copigmentation effects of phenolics on color enhancement and stability of blackberry wine residue anthocyanins: Chromaticity, kinetics and structural simulation. Food Chem..

[135] Maier T., Fromm M., Schieber A., Kammerer D.R., Carle R. (2009). Process and storage stability of anthocyanins and non-anthocyanin phenolics in pectin and gelatin gels enriched with grape pomace extracts. Eur. Food Res. Technol..

[136] Shi S., Lv M., Jin L., Qin G., Hao L. (2021). Antioxidant properties of anthocyanin revealed through the hydrogen atom transfer: Combined effects of temperature and pH. Mol. Phys..

[137] Xie C., Wang Q., Ying R., Wang Y., Huang M. (2019). Binding a chondroitin sulfate-based nanocomplex with kappa-carrageenan to enhance the stability of anthocyanins. Food Hydrocoll..

[138] Sadilova E., Stintzing F.C., Kammerer D.R., Carle R. (2009). Matrix dependent impact of sugar and ascorbic acid addition on color and anthocyanin stability of black carrot, elderberry and strawberry single strength and from concentrate juices upon thermal treatment. Food Res. Int..

[139] Oliveira H. (2020). Exploring the Applications of the Photoprotective Properties of Anthocyanins in Biological Systems. Int. J. Mol. Sci..

[140] Zhao R., Chen J., Yu S., Niu R., Yang Z., Wang H., Wang W. (2023). Active chitosan/gum Arabic-based emulsion films reinforced with thyme oil encapsulating blood orange anthocyanins: Improving multi-functionality. Food Hydrocoll..

[141] Zhao L., Pan F., Mehmood A., Zhang H., Ur Rehman A., Li J., Hao S., Wang C. (2021). Improved color stability of anthocyanins in the presence of ascorbic acid with the combination of rosmarinic acid and xanthan gum. Food Chem..

[142] Chung C., Rojanasasithara T., Mutilangi W., Mcclements D.J. (2016). Enhancement of colour stability of anthocyanins in model beverages by gum arabic addition. Food Chem..

[143] Fernandes A., Oliveira J., Fonseca F., Ferreira-Da-Silva F., Freitas V.D. (2019). Molecular binding between anthocyanins and pectic polysaccharides—Unveiling the role of pectic polysaccharides structure. Food Hydrocoll..

[144] Jiang Y., Yin Z., Wu Y., Qie X., He Z. (2021). Inhibitory effects of soy protein and its hydrolysate on the degradation of anthocyanins in mulberry extract. Food Biosci..

[145] Koh J., Xu Z., Wicker L. (2020). Blueberry pectin and increased anthocyanins stability under in vitro digestion. Food Chem..

[146] Kim I., Moon J.K., Sun J.H., Lee J. (2020). Structural Changes in Mulberry (*Morus Microphylla. Buckl*) and Chokeberry (*Aronia melanocarpa*) Anthocyanins during Simulated In Vitro Human Digestion. Food Chem..

[147] Ge J., Yue X., Wang S., Chi J., Liang J., Sun Y., Gao X., Yue P. (2019). Nanocomplexes composed of chitosan derivatives and β-Lactoglobulin as a carrier for anthocyanins: Preparation, stability and bioavailability in vitro. Food Res. Int..

[148] Wu Y., Han Y., Tao Y., Li D., Xie G., Show P.L., Lee S.Y. (2020). In vitro gastrointestinal digestion and fecal fermentation reveal the effect of different encapsulation materials on the release, degradation and modulation of gut microbiota of blueberry anthocyanin extract. Food Res. Int..

[149] Flores G., Luisa R., Costabile A., Klee A., Guergoletto K.B., Gibson G.R. (2015). In vitro fermentation of anthocyanins encapsulated with cyclodextrins: Release, metabolism and influence on gut microbiota growth. J. Funct. Foods.

[150] Han F., Yang P., Wang H., Fernandes I., Mateus N., Liu Y. (2019). Digestion and absorption of red grape and wine anthocyanins through the gastrointestinal tract. Trends Food Sci. Technol..

[151] Matera R., Gabbanini S., Berretti S., Amorati R., De Nicola G.R., Iori R., Valgimigli L. (2015). Acylated anthocyanins from sprouts of Raphanus sativus cv. Sango: Isolation, structure elucidation and antioxidant activity. Food Chem..

[152] Nile S.H., Park S.W. (2013). Edible berries: Bioactive components and their effect on human health. Nutrition.

[153] Azevedo J., Teixeira N., Oliveira J., Freitas V.D., Mateus N. (2011). Effect of sugar acylation on the antioxidant properties of Vitis vinifera red grape malvidinlucoside. Int. J. Food Sci. Technol..

[154] Zeng F., Zeng H.S., Ye Y., Zheng S., Zhuang Y., Liu J., Fei P. (2021). Preparation of acylated blueberry anthocyanins through an enzymatic method in an aqueous/organic phase: Effects on their colour stability and pH-response characteristics. Food Funct..

[155] Sariburun E., Sahin S., Demir C., Türkben C., Uylaşer V. (2010). Phenolic Content and Antioxidant Activity of Raspberry and Blackberry Cultivars. J. Food Sci..

[156] Vesna T.A., Gordana T.A., Jasna B., Pajin B., Djilas S. (2016). Sour cherry pomace extract encapsulated in whey and soy proteins: Incorporation in cookies. Food Chem..

[157] Liu R.H., Nehmer K.L. (2009). Cellular Antioxidant Activity (CAA) Assay. U.S. Patent.

[158] Villeneuve P. (2007). Lipases in lipophilization reactions. Biotechnol. Adv..

[159] Tan C., Celli G.B., Selig M.J., Abbaspourrad A. (2018). Catechin modulates the copigmentation and encapsulation of anthocyanins in polyelectrolyte complexes (pecs) for natural colorant stabilization. Food Chem..

[160] Grajeda-Iglesias C., Salas E., Barouh N., Barea B., Figueroa-Espinoza M.C. (2017). Lipophilization and MS characterization of the main anthocyanins purified from hibiscus flowers. Food Chem..

[161] Watson R.R. (2014). Polyphenols in Human Health and Disease.

[162] Lee J.Y., Jo Y.U., Shin H., Lee J., Chae S.U., Bae S.K., Na K. (2020). Anthocyanin-fucoidan nanocomplex for preventing carcinogen induced cancer: Enhanced absorption and stability. Int. J. Pharm..

[163] McGhie T.K., Walton M.C. (2007). The bioavailability and absorption of anthocyanins: Towards a better understanding. Mol. Nutr. Food Res..

[164] Chen M., Yu S. (2017). Lipophilized Grape Seed Proanthocyanidin Derivatives as Novel Antioxidants. J. Agric. Food Chem..

[165] Strugaa P.A., Dudra A., Gabrielska J. (2016). Interaction between Mimic Lipid Membranes and Acylated and Nonacylated Cyanidin and Its Bioactivity. J. Agric. Food Chem..

[166] Ryu D., Koh E. (2018). Stability of anthocyanins in bokbunja (*Rubus occidentalis* L.) under in vitro gastrointestinal digestion. Food Chem..

[167] Bars-Cortina D., Sakhawat A., Pinol-Felis C., Motilva M.J. (2022). Chemopreventive effects of anthocyanins on colorectal and breast cancer: A review. Semin. Cancer Biol..

[168] Peng Y., Yan Y., Wan P., Chen D., Ding Y., Ran L., Mi J., Lu L., Zhang Z., Li X. (2019). Gut microbiota modulation and anti-inflammatory properties of anthocyanins from the fruits of Lycium ruthenicum Murray in dextran sodium sulfate-induced colitis in mice. Free Radic. Biol. Med..

[169] Li S., Wang T., Wu B., Fu W., Xu B., Pamuru R.R., Kennett M., Vanamala J.K.P., Reddivari L. (2021). Anthocyanin-containing purple potatoes ameliorate DSS-induced colitis in mice. J. Nutr. Biochem..

[170] Li Z., Jiang H., Xu C., Gu L. (2015). A review: Using nanoparticles to enhance absorption and bioavailability of phenolic phytochemicals. Food Hydrocoll..

[171] Lagoa R., Silva J., Rodrigues J.R., Bishayee A. (2020). Advances in phytochemical delivery systems for improved anticancer activity. Biotechnol. Adv..

[172] Rahaiee S., Assadpour E., Faridi Esfanjani A., Silva A.S., Jafari S.M. (2020). Application of nano/microencapsulated phenolic compounds against cancer. Adv. Colloid Interface Sci..

[173] Liang T., Zhang Z., Jing P. (2019). Black rice anthocyanins embedded in self-assembled chitosan/chondroitin sulfate nanoparticles enhance apoptosis in HCT-116 cells. Food Chem..

[174] Szatrowski T.P., Nathan C.F. (1991). Production of Large Amounts of Hydrogen Peroxide by Human Tumor Cells. Cancer Res..

[175] Lee E.S., Na K., You H.B. (2003). Polymeric micelle for tumor pH and folate-mediated targeting. J. Control. Release.

[176] Lee E.S., Na K., Bae Y.H. (2005). Doxorubicin loaded pH-sensitive polymeric micelles for reversal of resistant MCF-7 tumor. J. Control. Release.

[177] Hamedi S., Koosha M. (2020). Designing a pH-responsive drug delivery system for the release of black-carrot anthocyanins loaded in halloysite nanotubes for cancer treatment. Appl. Clay Sci..

[178] Zhao L., Chen J., Wang Z., Shen R., Cui N., Sun A. (2015). Direct Acylation of Cyanidin-3-Glucoside with Lauric Acid in Blueberry and Its Stability Analysis. Int. J. Food Prop..

[179] Lívia D., Madalena D.A., Pinheiro A.C., Teixeira J.A., Vicente A.A., Ramos Ó. (2017). Micro- and nano bio-based delivery systems for food applications: In vitro behavior. Adv. Colloid Interface Sci..

[180] Tonon R.V., Brabet C., Hubinger M.D. (2010). Anthocyanin stability and antioxidant activity of spray-dried açai (Euterpe oleracea Mart.) juice produced with different carrier agents. Food Res. Int..

[181] Gamage G., Lim Y.Y., Choo W.S. (2022). Sources and relative stabilities of acylated and nonacylated anthocyanins in beverage systems. J. Food Sci. Technol..

[182] Tong Y., Deng H., Kong Y., Tan C., Chen J., Wan M., Wang M., Yan T., Meng X., Li L. (2020). Stability and structural characteristics of amylopectin nanoparticle-binding anthocyanins in *Aronia melanocarpa*. Food Chem..

